# Antidepressant effects of artemisinin and its derivatives: a systematic review and meta-analysis of randomized controlled animal studies

**DOI:** 10.3389/fphar.2026.1916381

**Published:** 2026-10-05

**Authors:** Yuan Tian, Haiping Zhang, Xu Zhang, Luyao Wang

**Affiliations:** 1 Sichuan Provincial Center for Mental Health, Sichuan Provincial People’s Hospital, School of Medicine, University of Electronic Science and Technology of China, Chengdu, China; 2 Key Laboratory of Psychosomatic Medicine, Chinese Academy of Medical Sciences, Chengdu, China

**Keywords:** animal experiment, artemisinin, depressive symptoms, meta-analysis, systematic review

## Abstract

**Introduction:**

Depression imposes a substantial global disease burden. Current pharmacotherapies are often limited by a delayed onset of action, significant adverse effects, and high costs. Preclinical studies have indicated that artemisinin and its derivatives possess neuroprotective and anti-inflammatory properties and have demonstrated antidepressant-like effects in rodent models. However, no meta-analysis has yet been performed on these preclinical rodent studies. This study aimed to systematically evaluate the antidepressant efficacy of artemisinin in rodent models of depression-like behavior and to identify potential factors influencing the variability in treatment effects.

**Methods:**

This study systematically searched four databases (PubMed, Web of Science, Embase, and China National Knowledge Infrastructure), including randomized controlled trials (RCTs) published up to May 2026. All data were statistically analyzed using a random-effects model, yielding standardized mean differences (SMDs) with their 95% confidence intervals (CIs). Subgroup analyses were conducted based on rodent species, depression-like modeling methods, intervention timing, duration, and frequency. Publication bias was assessed using Egger’s test, funnel plots, and trim-and-fill analyses. Sensitivity analyses were performed to evaluate result robustness. The risk of bias was evaluated using the Systematic Review Centre for Laboratory Animal Experimentation (SYRCLE) tool, and the overall certainty of evidence was evaluated using the Grading of Recommendations Assessment, Development and Evaluation (GRADE) framework.

**Results:**

A total of 17 studies involving 402 rodents were included in the analysis. Compared with the control group, artemisinin reduced immobility in the TST and FST, increased sucrose preference in the SPT, alleviated anxiety-like behaviors in the OFT and EPM, and improved cognitive performance in the MWM, Y-maze, and NOR. Subgroup analyses identified intervention duration, frequency, species, modeling methods, and derivative type as impactful moderators. Sensitivity tests validated stable outcomes. While Egger’s test hinted at publication bias in the OFT, TST, and FST datasets, trim-and-fill analyses imputed zero missing studies, although this does not fully exclude publication bias given the limited number of studies. Under the GRADE framework, the certainty of evidence was rated as very low for most outcomes due to high risk of bias, substantial heterogeneity, and indirectness.

**Discussion:**

Artemisinin can ameliorate depression-like and anxiety-like behaviors and improve cognitive function. These findings provide a basis for further investigation into artemisinin as an adjunctive therapeutic agent for depression. However, the certainty of evidence is very low due to methodological limitations in the included preclinical studies, and additional clinical trials are required to evaluate its efficacy in humans.

## 1 Introduction

Depression is a chronic, treatable psychiatric disorder with a lifetime prevalence of 15%–18% (Herrman et al., 2022) and a prevalence of 25% among individuals with chronic disorders (Berk et al., 2023). Patients with depression have a higher risk of premature mortality compared with the general population (Machado et al., 2018), imposing substantial medical and societal costs (Global, regional, and national burden of 12 mental disorders in 204 countries and territories, 1990-2019: a systematic analysis for the Global Burden of Disease Study, 2019., 2022). Although antidepressant therapy can effectively alleviate symptoms, it is frequently accompanied by various adverse effects, including nausea, insomnia, sexual dysfunction, metabolic abnormalities, and cardiovascular risk (Solmi et al., 2020). These adverse reactions significantly impair patients’ quality of life (Tandon et al., 2020), reduce treatment adherence, and increase healthcare costs (Whale et al., 2016). These challenges represent the current difficulties in depression treatment and avenues for the future.

Artemisinin is a natural sesquiterpene lactone compound extracted from Artemisia annua (Woodrow et al., 2005). The most well-established mechanism of action of artemisinin is related to its antimalarial activity, with its unique endoperoxide trioxane structure being critical for this effect (Klayman, 1985). In addition, artemisinin exhibits antitumor (Khanal, 2021), anti-inflammatory, and immunomodulatory effects (Xia et al., 2025). With the extensive exploration of artemisinin’s pharmacological activities, its antidepressant effects have been further investigated, with preclinical evidence suggesting that the antidepressant mechanism of artemisinin may be associated with anti-inflammatory and antioxidant actions (Huang et al., 2023). However, depression is not merely a neurotransmitter imbalance but also an immune-inflammatory-mediated systemic disease. Patients with depression exhibit significantly elevated levels of pro-inflammatory cytokines, such as IL-6 and TNF-α (Raison et al., 2006). Haonan Yu et al. designed and synthesized novel dihydroartemisinin-coumarin hybrids, demonstrating that these compounds can inhibit the release of NO, TNF-α, and IL-6 from activated microglial N9 cells, thereby reducing cancer-related fatigue syndrome associated with depressive mood in cancer patients (Yu et al., 2019). Animal experiments by Syeda Tayyaba Batool Kazmi further demonstrated that artemisinin compounds inhibit the NFκB/TNF-α/NLRP3 inflammatory pathway and activate the Nrf2/TRX antioxidant pathway in rodents, alleviating inflammatory injury and reducing depression-like and anxiety-like behaviors (Kazmi et al., 2024). However, a large-scale population-based study reported contrary findings, suggesting that antimalarial treatment may be a major independent risk factor for depression in children under 5 years of age in sub-Saharan Africa (Umeh and Adaji, 2025). Some studies have also reported adverse effects of artemisinin derivatives, including anxiety-like behaviors and insomnia, with artesunate being particularly associated with these effects compared to dihydroartemisinin (Grande et al., 2007). Furthermore, one review noted that, although observational and experimental evidence is extremely limited, pharmacological considerations suggest that artemisinin may theoretically have psychiatric adverse effects (Nevin and Croft, 2016). There have even been reports of artemisinin inducing mania in predisposed adolescents (Haq et al., 2009). Therefore, the antidepressant effect of artemisinin remains controversial.

A meta-analysis is a statistical method employed to systematically and quantitatively synthesize the results of multiple independent original studies that share a common research objective (Zeng et al., 2015). Currently, numerous animal experiments have investigated the ameliorative effects of artemisinin and its derivatives on depression-like behaviors in rodents. However, no meta-analysis has yet been conducted on these preclinical studies. Therefore, this study conducted a meta-analysis of preclinical research to evaluate the antidepressant efficacy of artemisinin and derivatives and to elucidate the underlying mechanisms of artemisinin and its derivatives in rodent models of depression-like.

## 2 Methods

### 2.1 Search strategy

A systematic literature search was conducted using Boolean operators across four electronic databases (PubMed, Web of Science, Embase, and China National Knowledge Infrastructure (CNKI)) with the search string “artemisinin AND depression” (Supplementary Table S7). The search period extended up to 1 May 2026. This systematic review and meta-analysis was registered with INPLASY (registration number INPLASY202680064).

### 2.2 Study selection

The titles and abstracts of the retrieved articles were screened in accordance with the PRISMA (Preferred Reporting Items for Systematic Reviews and Meta-Analyses) statement (Page et al., 2021). All retrieved records were initially screened independently by two researchers. Based on the titles and abstracts, the researchers conducted a preliminary assessment of the studies’ relevance, and records that did not meet the predefined inclusion criteria were excluded. For articles whose eligibility could not be determined definitively from the title and abstract alone, the full texts were retrieved and subjected to a detailed evaluation. In cases of disagreement between the two researchers during the screening process, a third researcher with relevant professional expertise was consulted to reach a consensus.

### 2.3 Inclusion criteria and exclusion criteria

Inclusion Criteria: Studies meeting the PICOS criteria were included in the analysis: (1) P (Population); Rodent models of depression-like behavior. (2) I (Intervention); Artemisinin and its derivatives were used as the sole intervention. (3) C (Control); Vehicle or placebo control, normal saline or an equivalent volume of solvent, subjected to the same handling procedures without active drug administration. (4) O (Outcomes); Depression-like behavior indicators, including the forced swimming test (FST), tail suspension test (TST), and sucrose preference test (SPT), anxiety-like behavior indicators, including the open field test (OFT) and elevated plus maze test (EPM), and cognitive function indicators, including the Morris water maze test (MWM), Y-maze test (Y-maze), and novel object recognition test (NOR). (5) S (Study design); Randomized controlled rodent studies.

Exclusion Criteria; (1) Abstracts, conference posters, reviews, meta-analyses, and case reports. (2) Studies lacking a group treated exclusively with artemisinin or its derivatives. (3) Studies with incomplete or unanalyzable data. (4) Studies not conducted in animal models.

### 2.4 Data extraction and synthesis

Two independent researchers screened the titles and abstracts to identify potentially relevant literature for this topic. After article selection, the reviewers analyzed the included studies and recorded the following details: study identifiers (author names, publication year, and country), characteristics (animal species, sex, and sample size), intervention features (derivative type, dosage, duration, and frequency of administration), and detailed outcome measures (FST, TST, OFT, SPT, MWM, EPM, Y-maze, and NOR). Subsequently, a third author compared the two databases and analyzed and resolved any inconsistencies in the extracted data.

### 2.5 Risk of bias assessment

We employed the SYRCLE tool to assess the risk of bias (ROB) (Hooijmans et al., 2014b). Two independent reviewers evaluated 10 core bias items, selection bias (random sequence generation, baseline characteristics, and allocation concealment); performance bias (random housing, blinding of caregivers and investigators), detection bias (random outcome assessment, blinding of outcome assessors), attrition bias (incomplete outcome data), reporting bias (selective outcome reporting), and other sources of bias. To assess potential publication bias across studies, a funnel plot analysis was conducted. Each study’s risk of bias was independently rated by the reviewers as “low,” “high,” or “unclear.” The overall certainty of evidence for each outcome (depression-like behaviors, anxiety-like behaviors, and cognitive function) was then assessed using the GRADE framework. We considered five downgrade criteria (risk of bias, inconsistency [I^2^ > 75%], indirectness, imprecision, and publication bias) and three upgrade criteria (large effect size, dose-response gradient, and plausible confounders), rating evidence certainty as high, moderate, low, or very low. Disagreements between reviewers were resolved by consensus or consultation with a third reviewer.

### 2.6 Statistical analyses

Data on means and standard deviations (SD) were extracted using Engauge Digitizer software (version 12.9.1). The numbers of animals in the intervention and control groups, along with the corresponding standard deviations (SD) of the outcomes, were extracted using Review Manager (version 5.4). Statistical analysis was performed using a random-effects model, and the generated forest plots displayed study-specific effect sizes with 95% confidence intervals (CIs) (González-Xuriguera et al., 2021). A P value of less than 0.05 was considered to indicate a statistically significant difference. Heterogeneity among the included studies was assessed using Higgins’ I^2^ statistic, categorized as low (25%), moderate (50%), or high (75%) (Higgins et al., 2003). To determine whether homogeneity existed across subgroups, the Chi^2^ test was also employed. Publication bias was evaluated using Egger’s regression test (Irwig et al., 1998). In addition, a sensitivity analysis was conducted by sequentially omitting each individual study to assess the robustness of the results. For studies reporting multiple behavioral outcomes from the same animal cohort, each outcome was analyzed in separate forest plots rather than pooled into a single composite estimate. Within-study correlation coefficients were not reported in the primary studies and could not be reliably estimated due to the limited number of studies per outcome, therefore, the precision of the pooled estimates may be marginally overestimated. To assess the robustness of our findings despite this limitation, we performed leave-one-out sensitivity analyses for all primary outcomes.

## 3 Results

### 3.1 Search and selection of studies

This systematic literature screening was strictly conducted in accordance with the PRISMA guidelines. The study retrieval process is illustrated in Figure 1. The search strategy encompassed four major databases: PubMed, Web of Science, Embase, and CNKI. The initial search yielded a total of 8,317 records. Following automated deduplication using EndNote, 68 duplicate records were excluded. Subsequently, 6,318 records that did not meet the inclusion criteria were removed (primarily due to being reviews or conference papers). Of the remaining 1,931 records, 1,799 studies were excluded after full-text analysis (mainly due to the absence of a depression-like model or the lack of artemisinin intervention). Ultimately, 132 articles met the eligibility criteria, of which 115 were excluded due to data issues (Supplementary Table S6). Consequently, 17 studies were included in the final analysis, with satisfactory consistency in the screening process. These studies were published between 2018 and 2025 and involved research institutions from two countries.

**FIGURE 1:**
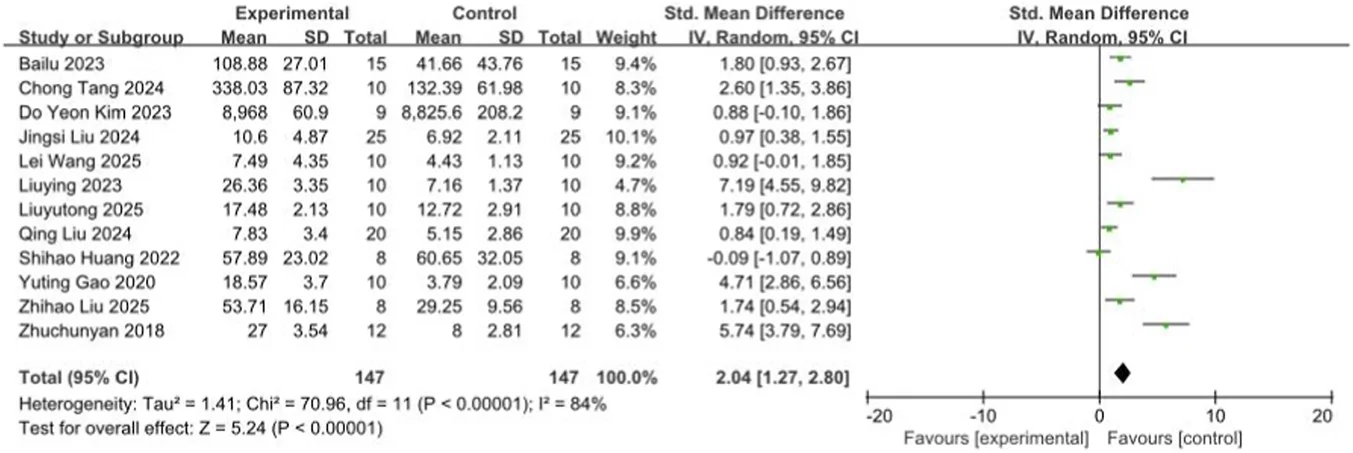
Flow diagram of literature screening.

### 3.2 Characteristics of the included studies

Supplementary Table S1 summarizes the characteristics of the 17 studies included in this systematic review. Among these, 16 studies (94.1%) were conducted in China and one in the Republic of Korea. The rodents were predominantly male, with a mean age of approximately 8 weeks and a mean body weight of approximately 20 g. 8 distinct modeling methods were employed to induce depression-like behaviors in the rodents, including spinal nerve ligation (SNL), chronic social defeat stress (CSDS), chronic unpredictable mild stress (CUMS), single prolonged stress (SPS), Ovariectomy (OVX), 3-Methylindole (3-MI), anti-N-methyl-D-aspartate receptor (Anti-NMDAR) and lipopolysaccharide-induced depression-like (LPS). The intervention dose, frequency, duration, and derivative type (artemisinin, dihydroartemisinin, or artesunate) of artemisinin and its derivatives were extracted for subsequent subgroup analyses. This geographic concentration and uniform sex distribution may constrain the external validity of the pooled estimates and limit generalizability across diverse research settings and patient populations.

### 3.3 Risk of bias assessment

The SYRCLE-based quality assessment revealed substantial methodological limitations across the included studies (Supplementary Table S2). While sequence generation, baseline characteristics, incomplete outcome data, and selective reporting were predominantly rated as low risk, critical domains prone to exaggerating treatment effects were frequently compromised. Notably, allocation concealment was universally rated as “unclear” across all 17 studies, precluding verification that group assignment was adequately shielded from investigators. Furthermore, random housing and random outcome assessment—key safeguards against confounding and selective measurement—were also universally classified as “unclear'. Regarding blinding, only 3 of 17 studies (17.6%) achieved adequate blinding of personnel and participants (performance bias), and while 11 studies were rated as low risk for blinding of outcome assessors, the remainder were either unclear or high risk. Under the GRADE framework, these methodological deficiencies contributed to an overall certainty of evidence rated as very low to low across all outcomes (Supplementary Table S4), with risk of bias serving as the principal driver for downgrading. Additional downgrading factors included substantial heterogeneity (I^2^ > 75%), indirectness inherent to preclinical rodent research, imprecision in cognitive outcomes, and suspected publication bias for TST, FST, and OFT. Specifically, the lack of allocation concealment and inadequate blinding collectively introduce a high probability of selection, performance, and detection biases, thereby threatening the internal validity of the pooled estimates and warranting cautious interpretation of the observed antidepressant effects.

### 3.4 Effects of artemisinin on depression-like behaviors

Using a random-effects model, a meta-analysis was conducted to evaluate the effects of artemisinin on various behavioral tests in rodent models of depression-like behaviors, including OFT, TST, FST, EPM, MWM, Y-maze, NOR, and SPT. The results are presented in the forest plot (Figures 2–4; Supplementary Figures S1, S2, S3, S4, S5). Depression-like behaviors (immobility time in TST and FST, and sucrose preference in SPT) were designated as the primary outcomes, while anxiety-like behaviors (time spent in the center of OFT and time spent in the closed arms of EPM) and cognitive function (escape latency in the MWM, latency to enter the novel arm in the Y-maze, and discrimination index in NOR) were considered secondary outcomes.

**FIGURE 2:**
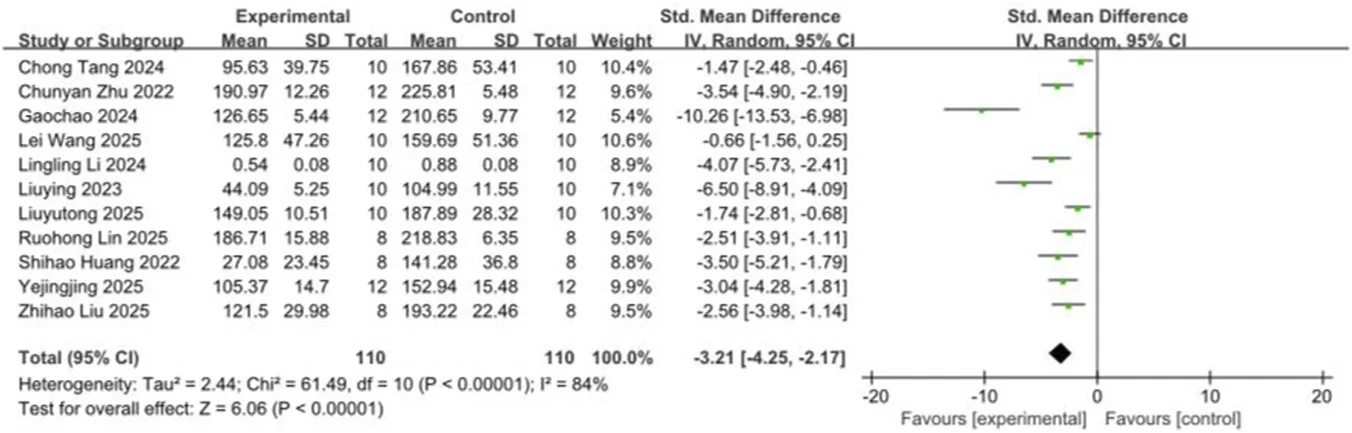
Forest plot of TST.

**FIGURE 3:**
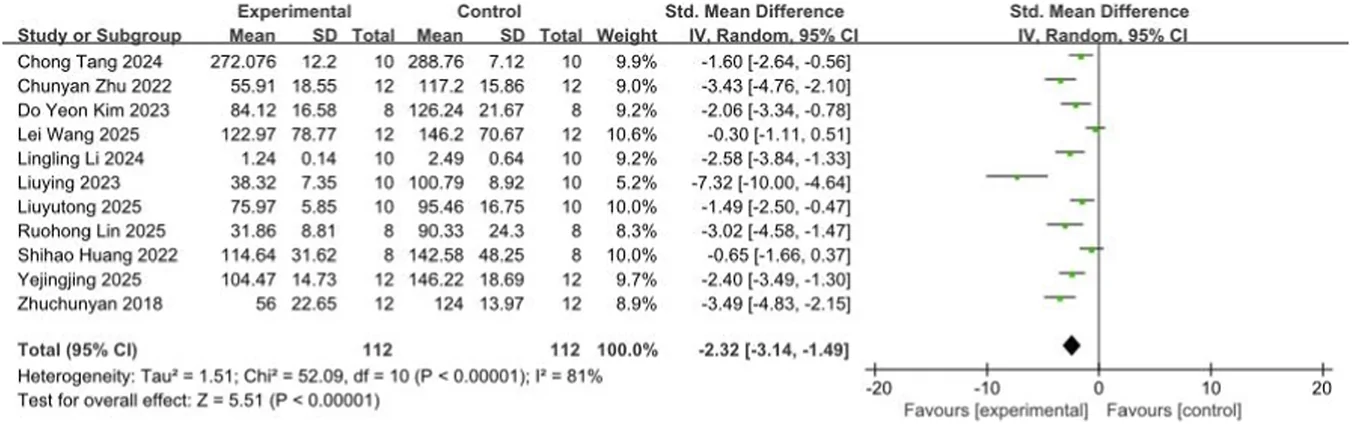
Forest plot of FST.

**FIGURE 4:**
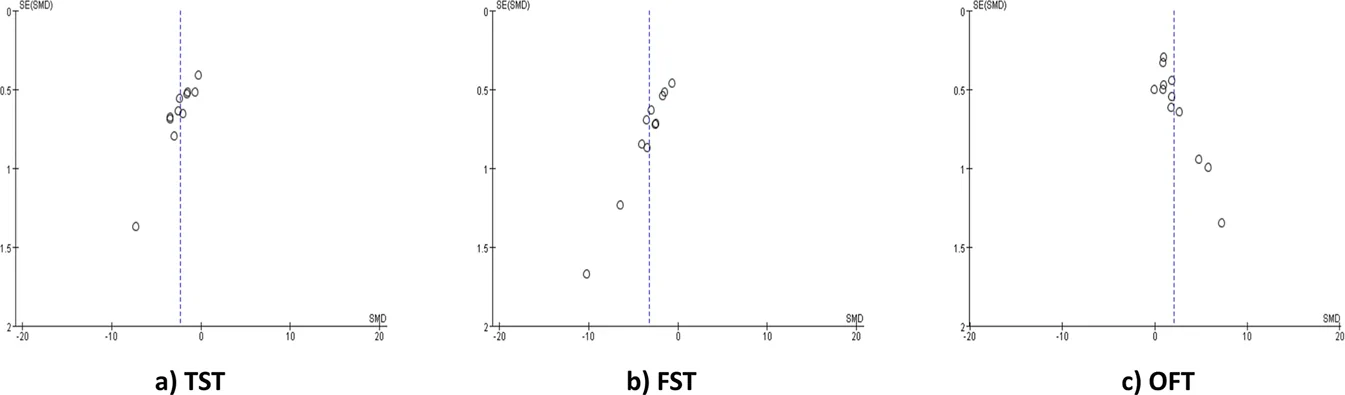
Funnel plots for assessment of publication bias. **(a)** TST, tail suspension test; **(b)** FST, forced swimming test; **(c)** OFT, open field test.

In the analysis of depression-like behaviors, a meta-analysis of 11 studies demonstrated that artemisinin and its derivatives significantly reduced immobility time in TST compared to the control group [SMD = −2.32, 95% CI = (−3.14, −1.49), P < 0.001] (Figure 2). High heterogeneity was observed among the included studies (I^2^ = 81%, P < 0.002). Similarly, a meta-analysis of 11 studies showed that artemisinin and its derivatives significantly decreased immobility time in FST relative to the control group [SMD = −3.21, 95% CI = (−4.25, −2.17), P < 0.001] (Figure 3), with high heterogeneity noted (I^2^ = 84%, P < 0.001). Furthermore, a meta-analysis of 8 studies revealed that artemisinin and its derivatives significantly increased sucrose preference in SPT compared to the control group [SMD = 2.45, 95% CI = (1.45, 3.45), P < 0.001], again with high heterogeneity detected among the included studies (I^2^ = 82%, P < 0.001) (Supplementary Figure S1).

### 3.5 Effects of artemisinin on anxiety-like behaviors

In anxiety-like behavior experiments, a meta-analysis of 12 studies revealed that artemisinin and its derivatives significantly increased the time spent in the center of OFT compared to the control group [SMD = 2.04, 95% CI = (1.27, 2.80), P < 0.001] (Figure 4). High heterogeneity was observed among the included studies (I^2^ = 84%, P < 0.001). Additionally, a meta-analysis of six studies demonstrated that artemisinin and its derivatives significantly reduced the time spent in the closed arms of EPM [SMD = −1.59, 95% CI = (−2.35, −0.83), P < 0.001], with high heterogeneity also noted across these studies (I^2^ = 76%, P < 0.001) (Supplementary Figure S2).

### 3.6 Effects of artemisinin and its derivatives on cognitive function

In cognitive function experiments, a meta-analysis of 3 studies revealed that artemisinin and its derivatives shortened the MWM escape latency compared with the control group [SMD = −4.72, 95% CI = (−9.04, −0.39), P < 0.01], with high heterogeneity observed among the included studies (I^2^ = 93%, P < 0.001) (Supplementary Figure S3), this estimate is not robust and should be viewed with due caution. A meta-analysis of 2 studies demonstrated that artemisinin and its derivatives reduced the latency to enter the novel arm in Y-maze compared with the control group [SMD = −2.60, 95% CI = (−4.69, −0.50), P = 0.02], with high heterogeneity observed among the included studies (I^2^ = 78%, P = 0.03) (Supplementary Figure S4). Another meta-analysis of 3 studies indicated that artemisinin and its derivatives significantly increased the NOR discrimination index compared with the control group [SMD = 2.13, 95% CI = (1.26, 3.00), P < 0.001], with no significant heterogeneity observed among the included studies (I^2^ = 49%, P = 0.14) (Supplementary Figure S5).

### 3.7 Subgroup analyses

#### 3.7.1 Depression-like behaviors

Subgroup analyses were performed according to the intervention dose, intervention cycle, intervention frequency, animal species (rodent type used for depression-like modeling), and depression-like modeling method to evaluate the effects of artemisinin and its derivatives on depression-like behavioral tests in animal models (Table 1). We caution that several subgroup comparisons were not mutually independent due to overlap in the constituent studies. For example, in the MWM analysis, the subgroup of studies using artemisinin (Supplementary Table S3) comprised the same single rat study that constituted the rat species subgroup (Table 1). Similarly, the longer-duration and stress-endocrine subgroups in MWM were derived from an identical set of studies. Consequently, apparent differences attributed to one moderator may be confounded by another, and the observed subgroup effects cannot be unequivocally attributed to any single factor. Therefore, these subgroup findings are exploratory and hypothesis-generating rather than confirmatory, and should be interpreted with caution.

**TABLE 1:** Subgroup analysis (the intervention dose, intervention cycle, intervention frequency, rodent type, and depression-like modeling method).

subgroup	df	I2	I2 P-val	Z	Z P-val	SMD	95%CI	subgroup P-val
​	1	​	​	​	​	​	​	0.04
OFT mice	7	87%	P < 0.001	4.79	P < 0.001	2.87	[1.70, 4.04]	​
OFT rats	1	67%	0.08	2.66	0.008	1.27	[0.33, 2.21]	​
​	1	​	​	​	​	​	​	0.08
OFT <100 mg/kg	5	91%	P < 0.001	3.86	0.0001	3.22	[1.59, 4.85]	​
OFT >100 mg/kg	3	55%	0.08	4.64	P < 0.001	1.64	[0.95, 2.34]	​
​	1	​	​	​	​	​	​	0.6
OFT once a day	7	82%	P < 0.001	4.93	P < 0.001	2.27	[1.37, 3.17]	​
OFT twice a day	1	95%	P < 0.001	1.27	0.21	3.94	[−2.15, 10.03]	​
​	1	​	​	​	​	​	​	0.98
OFT <2 weeks	3	85%	0.0001	3.33	0.0009	2.56	[1.05, 4.06]	​
OFT >2weeks	4	89%	P < 0.001	3.62	0.0003	2.58	[1.18, 3.98]	​
​	1	​	​	​	​	​	​	0.71
OFT stress and endocrinology	6	87%	P < 0.001	4.23	P < 0.001	2.6	[1.39, 3.80]	​
OFT immunity, inflammation, chemical damage	2	87%	0.0005	2.83	0.005	2.23	[0.68, 3.77]	​
​	1	​	​	​	​	​	​	0.99
TST mice	7	84%	P < 0.001	4.68	P < 0.001	−2.6	[−3.69, −1.51]	​
TST rats	0	​	​	4.05	P < 0.001	−2.58	[−3.84, −1.33]	​
​	1	​	​	​	​	​	​	0.14
TST <100 mg/kg	7	85%	P < 0.001	4.81	P < 0.001	−2.74	[−3.86, −1.62]	​
TST >100 mg/kg	0	​	​	3.02	0.002	−1.6	[−2.64, −0.56]	​
​	1	​	​	​	​	​	​	0.0004
TST once a day	7	77%	P < 0.001	5.14	P < 0.001	−2.21	[−3.05, −1.36]	​
TST twice a day	0	​	​	5.36	P < 0.001	−7.32	[−10.00, −4.64]	​
​	1	​	​	​	​	​	​	0.22
TST <2weeks	2	88%	0.0003	1.96	P < 0.00001	−1.73	[−3.45, −0.00]	​
TST >2weeks	5	73%	0.002	5.52	P < 0.00001	−3	[−4.07, −1.94]	​
​	1	​	​	​	​	​	​	0.4
FST mice	7	87%	P < 0.001	4.66	P < 0.001	−3.15	[−4.48, −1.83]	​
FST rats	0	​	​	4.81	P < 0.001	−4.07	[−5.73, −2.41]	​
​	1	​	​	​	​	​	​	0.06
FST <100 mg/kg	7	88%	P < 0.001	4.97	P < 0.001	−3.62	[−5.05, −2.19]	​
FST >100 mg/kg	1	34%	0.22	3.56	0.0004	−1.9	[−2.94, −0.85]	​
​	1	​	​	​	​	​	​	0.007
FST once a day	8	83%	P < 0.001	5.27	P < 0.001	−2.87	[−3.94, −1.80]	​
FST twice a day	0	​	​	5.28	P < 0.001	−6.5	[−8.91, −4.09]	​
​	1	​	​	​	​	​	​	0.0006
FST <2weeks	1	27%	0.24	2.55	0.01	−1.03	[−1.82, −0.24]	​
FST >2weeks	6	83%	P < 0.001	5.36	P < 0.001	−3.91	[−5.34, −2.48]	​
​	1	​	​	​	​	​	​	0.83
EPM mice	1	90%	0.001	1.51	0.13	−1.93	[−4.42, 0.57]	​
EPM rats	2	81%	0.005	2.66	0.008	−1.62	[−2.81, −0.43]	​
​	1	​	​	​	​	​	​	0.1
EPM <100 mg/kg	2	87%	0.0004	2.55	0.01	−2.51	[−4.44, −0.58]	​
EPM >100 mg/kg	1	0%	0.63	3.52	0.0004	−0.82	[−1.28, −0.36]	​
​	1	​	​	​	​	​	​	0.06
EPM once a day	3	83%	0.0005	3.27	0.001	−2.02	[−3.23, −0.81]	​
EPM twice a day	0	​	​	2.51	0.01	−0.74	[−1.31, −0.16]	​
​	1	​	​	​	​	​	​	0.67
EPM <2weeks	1	87%	0.005	1.77	0.08	−2.05	[−4.32, 0.22]	​
EPM >2weeks	2	83%	0.002	2.57	0.01	−1.5	[−2.65, −0.36]	​
​	1	​	​	​	​	​	​	0.01
EPM stress and endocrinology	0	​	​	5.73	P < 0.001	−3.65	[−4.90, −2.40]	​
EPM immunity, inflammation, chemical damage	2	81%	0.005	2.55	0.01	−1.47	[−2.60, −0.34]	​
​	1	​	​	​	​	​	​	0.37
MWM mice	1	86%	0.007	1.26	0.21	−3.67	[−9.37, 2.03]	​
MWM rats	0	​	​	5.28	P < 0.001	−6.5	[−8.91, −4.09]	​
​	1	​	​	​	​	​	​	0.28
MWM once a day	1	94%	P < 0.001	1.37	0.17	−3.69	[−8.96, 1.59]	​
MWM twice a day	0	​	​	5.32	P < 0.001	−6.89	[−9.44, −4.35]	​
​	1	​	​	​	​	​	​	P < 0.001
MWM <2weeks	0	​	​	2.27	0.02	−1.11	[−2.07, −0.15]	​
MWM >2weeks	1	0%	0.82	7.49	P < 0.001	−6.69	[−8.44, −4.94]	​
​	1	​	​	​	​	​	​	P < 0.001
MWM stress and endocrinology	1	0%	0.82	7.49	P < 0.001	−6.69	[−8.44, −4.94]	​
MWM immunity, inflammation, chemical damage	0	​	​	2.27	0.02	−1.11	[−2.07, −0.15]	​
​	1	​	​	​	​	​	​	0.002
SPT mice	4	77%	0.002	4.16	P < 0.001	2.51	[1.33, 3.70]	​
SPT rats	0	​	​	5.88	P < 0.001	6.49	[4.33, 8.65]	​
​	1	​	​	​	​	​	​	0.02
SPT <100 mg/kg	3	86%	0.0001	3.69	0.0002	3.77	[1.76, 5.77]	​
SPT >100 mg/kg	2	27%	0.25	3.95	P < 0.001	1.33	[0.67, 1.99]	​
​	1	​	​	​	​	​	​	0.06
SPT <2weeks	1	63%	0.1	2.46	0.01	1.37	[0.28, 2.46]	​
SPT >2weeks	4	85%	P < 0.001	3.91	P < 0.001	3.24	[1.61, 4.86]	​
​	1	​	​	​	​	​	​	0.02
SPT stress and endocrinology	4	69%	0.01	4.63	P < 0.001	2.37	[1.37, 3.37]	​
SPT immunity, inflammation, chemical damage	0	​	​	2.3	0.02	0.89	[0.13, 1.64]	​

Open Field Test: OFT; Tail Suspension Test: TST; Forced Swim Test: FST; Elevated Plus Maze Test: EPM; Morris Water Maze Test: MWM; Y-maze test: Y-maze; Novel Object Recognition Test: NOR; Sucrose Preference Test: SPT.

In the depression-like behavioral experiments, data from TST across nine studies indicated that an intervention frequency of twice daily (Standardized Mean Difference [SMD] = −7.32, 95% Confidence Interval [CI] = [−10.00, −4.64]) was more effective in reducing immobility time than a once-daily regimen (SMD = −2.21, 95% CI = [−3.05, −1.36]), despite significant heterogeneity (I^2^ = 92.9%, P < 0.001). However, the twice-daily subgroup comprised only one study, therefore, no reliable comparison can be made, and any apparent difference should be regarded as hypothesis-generating only. Data from FST across ten studies demonstrated that a twice-daily intervention frequency (SMD = −6.50, 95% CI = [−8.91, −4.09]) was also superior to a once-daily frequency (SMD = −2.87, 95% CI = [−3.94, −1.80]) in reducing immobility time, notwithstanding significant heterogeneity (I^2^ = 86.3%, P < 0.05). As this comparison was based on only one study in the twice-daily subgroup, this finding is preliminary and requires confirmation in additional studies. Furthermore, FST data from nine studies revealed that an intervention cycle longer than 2 weeks (SMD = −3.91, 95% CI = [−5.34, −2.48]) was more effective than a cycle shorter than 2 weeks (SMD = −1.03, 95% CI = [−1.82, −0.24]) in decreasing immobility time, albeit with significant heterogeneity (I^2^ = 91.6%, P < 0.001). The short-cycle subgroup comprised only two studies, limiting the reliability of this comparison. In SPT, data from seven studies showed that rats (SMD = 6.49, 95% CI = [4.33, 8.65]) exhibited a greater increase in sucrose preference compared to mice (SMD = 2.51, 95% CI = [1.33, 3.70]), despite significant heterogeneity (I^2^ = 90.0%, P = 0.002). However, the rat subgroup comprised only one study, therefore, no reliable comparison can be made, and any apparent difference should be regarded as hypothesis-generating only. Regarding the depression-like modeling method, models established using stress-endocrine approaches (SMD = 2.37, 95% CI = [1.37, 3.37]) demonstrated a greater response to artemisinin intervention than those based on immune-inflammatory or chemical injury methods (SMD = 0.89, 95% CI = [0.13, 1.64]), although significant heterogeneity was observed (I^2^ = 81.3%, P = 0.02). As the immune-inflammatory/chemical injury subgroup was represented by only one study, this comparison is exploratory and hypothesis-generating rather than conclusive. No statistically significant differences were detected in the remaining subgroup analyses (P > 0.05). Given the small number of studies in each subgroup, this difference should be interpreted cautiously as a preliminary observation rather than a definitive conclusion.

#### 3.7.2 Anxiety-like behaviors

In the anxiety-like behavior experiments, data from ten studies using OFT showed that mice (SMD = 2.87, 95% CI: [1.70, 4.04]) exhibited a greater increase in the time spent in the central area of the OFT compared to rats (SMD = 1.27, 95% CI; [0.33, 2.21]), despite significant heterogeneity (I^2^ = 76.9%, P = 0.04). No statistically significant differences were observed in the remaining subgroups (P > 0.05). Notably, the rat subgroup was represented by only two studies; thus, this subgroup comparison is exploratory and requires validation in larger samples.

#### 3.7.3 Cognitive function

In cognitive function experiments, data from three studies using MWM indicated that artemisinin treatment lasting more than 2 weeks (SMD = −6.69, 95% CI = [−8.44, −4.94]) significantly reduced MWM escape latency compared to treatment durations shorter than 2 weeks (SMD = −1.11, 95% CI = [−2.07, −0.15]), despite substantial heterogeneity (I^2^ = 96.7%, P < 0.001). Furthermore, MWM data from three studies revealed that depression-like rodent models established through stress-related endocrine methods (SMD = −6.69, 95% CI = [−8.44, −4.94]) exhibited a more favorable response to artemisinin intervention than those induced by immune-inflammatory or chemical injury methods (SMD = −1.11, 95% CI = [−2.07, −0.15]), again with notable heterogeneity (I^2^ = 81.3%, P = 0.02). Notably, in this dataset, the longer-duration subgroup and the stress-endocrine subgroup comprised the same set of studies, thus, these subgroup comparisons are not independent. No statistically significant differences were observed among the remaining subgroups (P > 0.05). Given that this comparison was based on a total of only three studies, with one subgroup represented by a single study, these findings are highly exploratory and should not be regarded as definitive evidence.

#### 3.7.4 Subgroup analysis by artemisinin derivative type

Exploratory subgroup analyses were conducted to compare the antidepressant, anxiolytic, and cognitive-enhancing effects of three artemisinin derivatives, dihydroartemisinin, artesunate, and artemisinin (Supplementary Table S3). Specifically, Artemisia annua extract (Do Yeon et al., 2023) and absinthium L. essential oil (Liu et al., 2025) were excluded from the derivative-type subgroup analysis because they could not be classified into the three pure derivative categories (artemisinin, dihydroartemisinin, or artesunate). The study by Gaochao et al. (2024) using artemisinin ester was classified under artesunate, as artesunate represents the hemisuccinate ester of dihydroartemisinin, *,* as both compounds share a similar esterified structural modification and analogous pharmacokinetic properties, however, we acknowledge this is a pragmatic classification and have noted this limitation. In the OFT, dihydroartemisinin demonstrated a significantly greater effect on increasing central area time (SMD = 3.67, 95% CI [2.21, 5.13], P < 0.001) compared with artesunate (SMD = 0.51, 95% CI: [−0.52, 1.54], P = 0.33) and artemisinin (SMD = 0.86, 95% CI [0.33, 1.40], P = 0.001), with a statistically significant subgroup difference (P = 0.0009). In the MWM, artemisinin significantly reduced escape latency (SMD = −6.50, 95% CI: [−8.91, −4.09], P < 0.001) compared with dihydroartemisinin (SMD = −3.88, 95% CI [–9.54, 1.79], P = 0.18), with a significant subgroup difference (P = 0.04). No statistically significant subgroup differences were observed for TST (P = 0.32), FST (P = 0.32), EPM (P = 0.33), NOR (P = 0.86), or SPT (P = 0.20). Across all behavioral domains, the direction of the treatment effect was generally consistent across the three derivatives. However, these comparisons are highly exploratory: several subgroups were represented by only one or two studies, resulting in wide confidence intervals and limited statistical power. Consequently, any apparent differences in efficacy among artemisinin derivatives should be interpreted with extreme caution and require confirmation in adequately powered, head-to-head comparisons.

### 3.8 Publication bias and sensitivity analysis

When individual studies were sequentially excluded and the meta-analysis was repeated, the pooled effect sizes for the FST, TST, and OFT remained within their respective confidence intervals, and the statistical significance was preserved (Figure 5). These findings indicate that artemisinin and its derivatives reliably ameliorate depression-like and anxiety-like behaviors in mice and that this effect was not disproportionately influenced by any single study, despite the presence of substantial heterogeneity. However, the P values from both Egger’s test were less than 0.05 (Table 2), and the funnel plot exhibited asymmetry, suggesting a potential publication bias (Figure 6). Interpretation of these findings requires caution. With relatively few studies in each analysis, funnel plots and Egger’s regression tests have limited statistical power and wide confidence intervals, reducing their reliability. Moreover, funnel plot asymmetry reflects small-study effects—a composite phenomenon in which smaller studies yield systematically different effect estimates than larger ones. Such asymmetry may arise from multiple sources, including true clinical or methodological heterogeneity, lower methodological quality in smaller studies, selective outcome reporting, and/or publication bias. Therefore, we cannot reliably distinguish true publication bias from other small-study effects, such as genuine between-study heterogeneity, differential methodological rigor, or selective reporting within individual experiments. This uncertainty further tempers the certainty of the pooled estimates. To further evaluate whether publication bias substantially influenced the pooled estimates, we conducted trim-and-fill analyses. The trim-and-fill procedure imputed zero missing studies for TST, FST, and OFT, indicating that the observed asymmetry in funnel plots likely reflects true heterogeneity rather than publication bias (Supplementary Table S5). However, this result does not exclude the possibility of publication bias, particularly given the small number of studies and the limited statistical power of funnel-plot-based methods in preclinical meta-analyses. The observed asymmetry may still reflect small-study effects, selective reporting, or true heterogeneity, and the pooled effect sizes should therefore be interpreted with caution.

**FIGURE 5:**
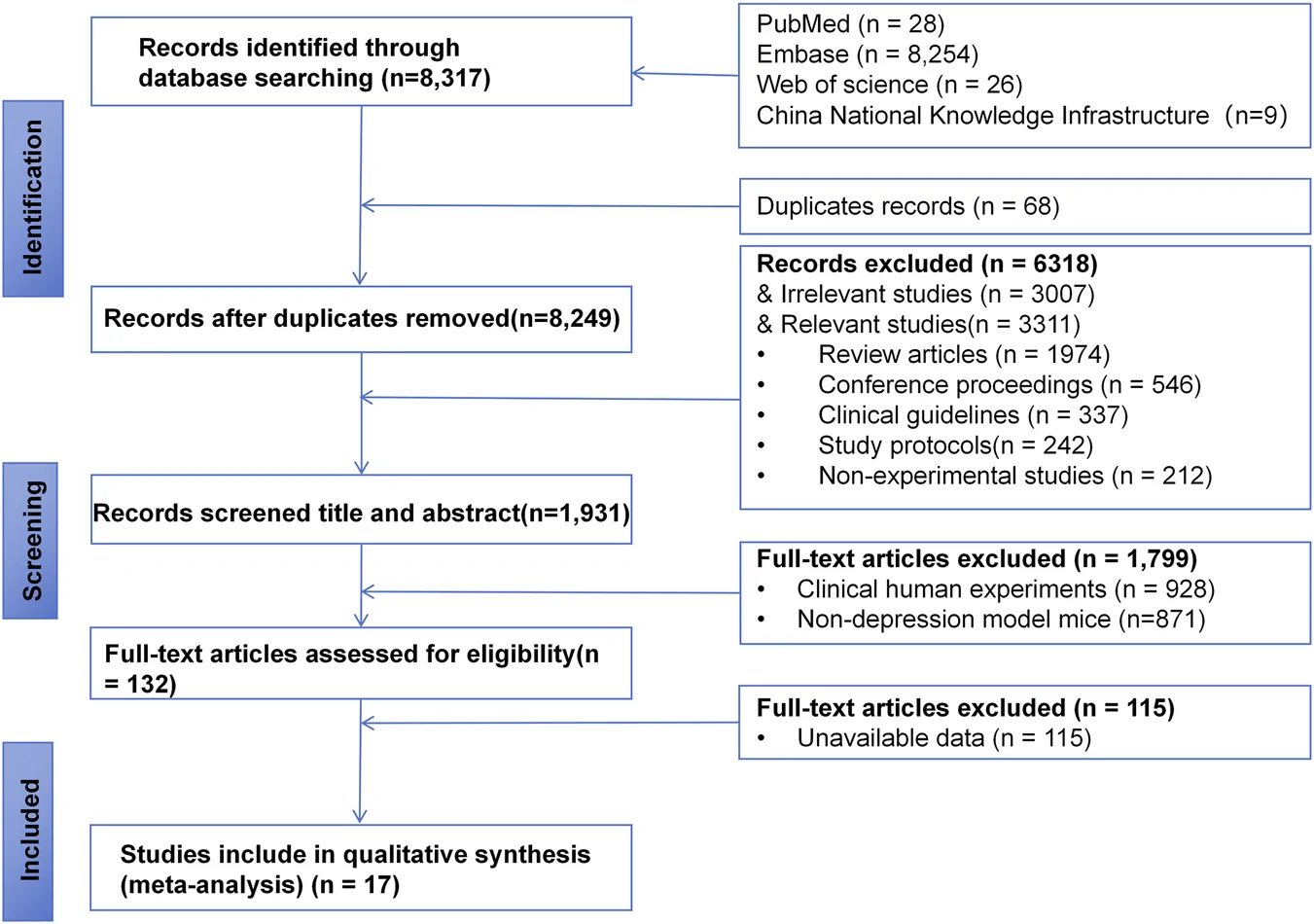
Sensitivity analysis.

**TABLE 2:** Egger’s test.

TST	​	​	​	​	​
Std_Eff	Coef	Std. Err	t	P>|t|	[95% conf. Interval]
Slope	2.054756	0.353,802	5.81	0	1.2544 2.855,112
Bias	−5.923,094	0.5,420,584	−10.93	0	−7.149,315, −4.696,873
FST	​	​	​	​	​
Std_Eff	Coef	Std. Err	t	P>|t|	[95% conf. Interval]
Slope	1.619,184	0.2,638,618	6.14	0	1.022287 2.216,081
Bias	−5.249,093	0.3,327,624	−15.77	0	−6.001853, −4.496,332
OFT	​	​	​	​	​
Std_Eff	Coef	Std. Err	t	P>|t|	[95% conf. Interval]
Slope	−0.6,821,209	0.4,898,058	−1.39	0.194	−1.773,476, 0.4,092,344
Bias	4.175,316	0.8,445,099	4.94	0.001	2.29363, 6.057001

**FIGURE 6:**
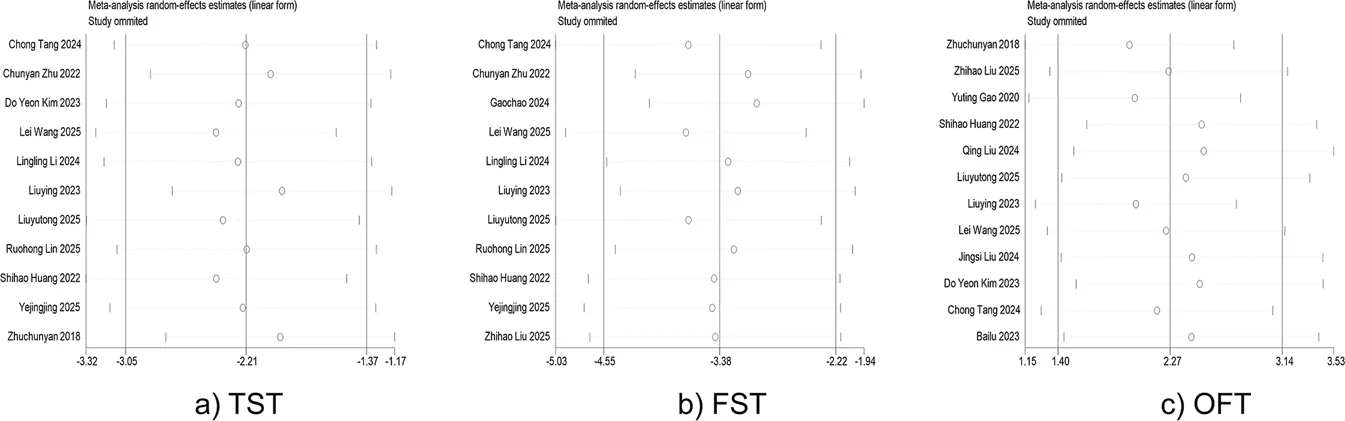
Funnel plot on publication bias. **(a)** TST. **(b)** FST. **(c)** OFT.

## 4 Discussion

It is critical to distinguish the scope of the present meta-analysis from the mechanistic interpretations discussed herein. This meta-analysis quantitatively synthesized behavioral outcomes (FST, TST, SPT, OFT, EPM, MWM, Y-maze, and NOR) and did not directly evaluate molecular, cellular, or histopathological mechanisms. The mechanistic explanations presented below are therefore derived from the individual primary studies included in this review and from the broader preclinical literature, rather than being established by the pooled quantitative synthesis itself.

Our study represents the first meta-analysis to investigate the effects of artemisinin and its derivatives on depression-like behaviors in rodents. This meta-analysis, encompassing 17 published studies, aimed to evaluate the preclinical evidence regarding the efficacy of artemisinin and its derivatives in ameliorating depression-like behaviors in rodent models. Our findings demonstrated that artemisinin and its derivatives exerted beneficial effects on depression-like behaviors in animal models. These effects included a reduction in immobility time in FST and TST, indicating improvement in despair-like behavior, as well as an increase in sucrose consumption in SPT, reflecting an amelioration of anhedonia. Furthermore, improvements were observed in anxiety-like behaviors, evidenced by an increased time spent in the central area during OFT and a decreased time spent in the closed arms of EPM. Cognitive function was also enhanced, as demonstrated by a reduction in escape latency in MWM, a decreased latency to enter the novel arm in Y-maze, and an increased discrimination index in NOR. Subgroup analyses further indicated that these therapeutic effects were influenced by intervention frequency, intervention duration, rodent species, the type of modeling method employed, and derivative type.

It is imperative to interpret these findings in light of the considerable risk of bias identified across the included studies. The SYRCLE assessment demonstrated that no study adequately reported allocation concealment, and the vast majority failed to implement or report blinding of caregivers and investigators. In preclinical research, inadequate allocation concealment increases the risk of baseline imbalances and selective group assignment, while lack of blinding is known to inflate effect sizes in behavioral outcomes. Consequently, the magnitude of the observed beneficial effects of artemisinin and its derivatives may be overestimated, and the true effect size in rigorously controlled experiments may be more modest than reported herein. Under the GRADE framework, these methodological deficiencies contributed to an overall certainty of evidence rated as very low to low across all outcomes (Supplementary Table S4), with risk of bias serving as the principal driver for downgrading. Additional downgrading factors included substantial inconsistency (I^2^ > 75% for most outcomes), serious indirectness arising from the exclusive use of rodent models and limited demographic diversity (94.1% of studies from China, predominantly male animals), imprecision in cognitive function and anxiety-like subgroups with smaller study numbers, and suspected publication bias for primary depression and anxiety-like outcomes (TST, FST, and OFT). Therefore, the pooled effect sizes should be interpreted as potentially inflated estimates rather than precise quantifications of efficacy, and the antidepressant effects observed in these rodent models may not replicate under methodologically stringent conditions.

Our meta-analysis confirmed that artemisinin and its derivatives reduce depression-like behaviors in rodent models. The biological mechanisms underlying these behavioral improvements have been proposed in the primary literature. Ruohong Lin’s animal experiments demonstrated that artemisinin exerts significant antidepressant effects in the chronic unpredictable mild stress (CUMS) model (Lin et al., 2025). Specifically, artemisinin activates the AKT and ERK signaling pathways, thereby upregulating BDNF/TrkB/ERK/CREB and AKT/GSK/NRF2/HO1 cascades, which in turn modulate astrocyte activity and promote hippocampal neurogenesis, ultimately conferring neuroprotective effects. Meanwhile, dihydroartemisinin ameliorates chronic stress-induced depression-like behaviors by reshaping gut microbiota balance and inhibiting hippocampal neuroinflammation via the gut-brain axis, while also increasing BDNF levels (Tang et al., 2024). In addition, artemisinin modulates GABAergic neurotransmission in a dose-dependent manner, whereas low doses of artemisinin enhance GABAergic signaling (Xia et al., 2024). Some researchers have covalently hybridized artemisinin derivatives with gamma-aminobutyric acid (GABA) to design antidepressant compounds with improved lipophilicity, as well as antioxidant and anti-inflammatory properties, combined with the inhibitory neuromodulatory function of GABA (He et al., 2021). The antidepressant mechanisms of artemisinin may involve the activation of distinct signaling pathways and the modulation of neurotransmitters, thereby exerting anti-inflammatory, antioxidant, and neuroprotective effects. These mechanistic pathways, though consistent with the behavioral improvements observed in our pooled analysis, were not directly evaluated or quantitatively synthesized in the present meta-analysis.

Our meta-analysis demonstrated that artemisinin and its derivatives reduce anxiety-like behaviors across multiple behavioral paradigms. Individual studies have suggested that these behavioral effects may involve antioxidant and anti-inflammatory mechanisms. For example, Huang et al. reported that artesunate attenuates anxiety-like symptoms through antioxidant properties in a lipopolysaccharide-induced mouse model (Huang et al., 2023). Furthermore, Kazmi et al. elucidated that artemisinin exerts dose-dependent anti-inflammatory effects via dual activation of the Nrf2/TRX antioxidant pathway and inhibition of the NF-κB/TNF-α/NLRP3 inflammatory pathway. At a dose of 100 mg/kg, a significant reduction in DNA damage was observed (Kazmi et al., 2024). Although different types of artemisinin derivatives exhibit varying degrees of influence on anxiety-like emotions, they may concurrently ameliorate anxiety-like and depression-like symptoms associated with neuropathic pain through anti-inflammatory and antioxidant mechanisms (Zhu et al., 2018). Consistent with these findings, our meta-analysis demonstrated that artemisinin and its derivatives alleviate anxiety-like behaviors induced by various stimuli. It must be emphasized that these mechanistic insights originate from individual experiments and were not subject to quantitative pooling in the present study.

Our meta-analysis indicated that artemisinin and its derivatives improve cognitive performance in rodent models. Several mechanisms have been hypothesized in the literature to explain this observation. Artemisinin has been reported to exert neuroprotective effects by reducing oxidative stress (Lu et al., 2019). And Zhao et al. showed that its anti-inflammatory effects may involve modulation of the TLR4/NF-κB signaling pathway, attenuating Aβ-induced neuroinflammation (Zhao et al., 2022). However, even when depression symptoms are alleviated, cognitive deficits may persist and adversely affect various aspects of daily life, thereby significantly reducing quality of life (Czerwińska and Pawłowski, 2020). Furthermore, based on its characteristics—including the peroxide bridge-iron ion-free radical mechanism, potent anti-malarial activity, anti-inflammatory effects, and neuroprotection—artemisinin has been demonstrated to be effective in cerebral malaria (de Miranda et al., 2011). However, because our meta-analysis was restricted to behavioral readouts, these mechanistic explanations remain speculative and require direct experimental validation.

Our meta-analysis identified that a twice-daily intervention frequency and a duration exceeding 2 weeks were associated with greater behavioral improvements. The optimal dose for mood regulation remains unclear. Lin et al. suggested a potential inverted U-shaped dose-response curve, with 1 mg/kg potentially being the optimal dose (Lin et al., 2025). Whereas Huang et al. reported efficacy at 5–15 mg/kg [12], although further refinement to determine the optimal dose was not conducted. The inconsistency among these findings necessitates additional experiments to clarify the optimal intervention dose, frequency, and duration of artemisinin and its derivatives. Furthermore, the therapeutic efficacy of artemisinin and its derivatives was influenced by rodent species and the method of depression-like induction. In depression-like behavior tests, rats showed a better response to artemisinin and its derivatives intervention, whereas in anxiety-like behavior tests, mice exhibited a better response. This discrepancy may be attributed not only to differences in rodent species but also to variations in inbred strains, as well as environmental factors such as light intensity and circadian rhythms (Archer, 1973). In SPT and MWM, depression-like models induced by stress-endocrine methods demonstrated greater responsiveness to artemisinin intervention compared to those induced by immune-inflammatory or chemical injury methods. This may be because the antidepressant mechanisms of artemisinin-like compounds are highly compatible with the pathological features of stress models, whereas inflammatory models are limited by factors such as dosing strategies, pathological severity, and mechanistic mismatch. This hypothesis is supported by the study of Xiaojin Yu et al., who compared different depression-like modeling methods and found that inflammatory depression-like models inherently exhibited lower responsiveness to antidepressant drugs (Yu et al., 2022). These dose-response observations are derived from subgroup analyses of behavioral data and should not be interpreted as mechanistic conclusions.

Exploratory subgroup analyses further suggested that the magnitude of artemisinin’s effects may vary by derivative type (Supplementary Table S3). Dihydroartemisinin appeared to exert superior anxiolytic effects in the OFT, whereas artemisinin demonstrated greater efficacy in improving cognitive performance in the MWM. However, these comparisons were based on very small subgroup sizes and should be regarded as hypothesis-generating rather than definitive. While our subgroup analyses identified several potential moderators of artemisinin’s antidepressant effects, including intervention frequency, treatment duration, rodent species, depression-like models, and derivative type, we emphasize that these findings are exploratory and hypothesis-generating rather than definitive. Several subgroups were represented by a very small number of studies (in some cases, only one or two), resulting in wide confidence intervals and limited statistical power to detect true differences. In such instances, the observed subgroup differences may reflect random variation rather than genuine biological or methodological effects. Consequently, conclusions regarding the “optimal” intervention frequency, treatment duration, most responsive animal species, depression-like models or derivative types should be interpreted with considerable caution. These subgroup findings are best viewed as preliminary insights to inform the design of future primary studies, rather than as evidence-based recommendations for experimental practice. Future preclinical research should prioritize head-to-head comparisons within standardized protocols to more rigorously evaluate these potential moderators.

The substantial heterogeneity observed in this meta-analysis, with I^2^ values frequently exceeding 80%, is consistent with the inherent nature of preclinical research, where animal studies are generally smaller and substantially more heterogeneous than clinical trials due to diversity in species, strains, induction models, and intervention protocols (Hooijmans et al., 2014a; Vesterinen et al., 2014). However, I^2^ should not be interpreted in isolation as an absolute measure of heterogeneity, as it is a proportion rather than an absolute amount, and its magnitude depends heavily on study precision and sample size; consequently, a high I^2^ may reflect substantial true variation or only modest between-study variance, depending on the context (Ioannidis et al., 2007; Borenstein et al., 2017). In the present study, the observed heterogeneity can be attributed to several clinically and methodologically plausible sources, including eight distinct depression-like models with divergent pathophysiological mechanisms (stress-endocrine versus immune-inflammatory versus chemical injury), different artemisinin derivatives and dosing regimens, and species differences between rats and mice, all of which were partially corroborated by our subgroup analyses (Hooijmans et al., 2014a; Vesterinen et al., 2014). Despite this variability, the direction of the treatment effect remained remarkably consistent across the vast majority of included studies, with artemisinin consistently ameliorating depression-like, anxiety-like, and cognitive outcomes, suggesting a true biological effect rather than random noise (Higgins et al., 2003). Furthermore, the stability of our pooled estimates in leave-one-out sensitivity analyses, combined with the use of a random-effects model that explicitly incorporates between-study variance and provides more conservative confidence intervals, supports the robustness of our conclusions (Riley et al., 2011; Hooijmans et al., 2014a). Importantly, rather than undermining the validity of our findings, this heterogeneity may reflect genuine biological and environmental variability across experimental conditions; indeed, preclinical research has shown that more representative and heterogeneous study samples improve external validity and reproducibility compared with highly standardized single-laboratory designs (Voelkl et al., 2018). It is noteworthy that variability in artemisinin derivative type (artemisinin, dihydroartemisinin, and artesunate) also contributed to between-study heterogeneity; however, given the small number of studies within each derivative subgroup, these apparent differences should be interpreted as exploratory rather than definitive.

The funnel plot asymmetry and significant Egger’s test for TST, FST, and OFT indicate potential publication bias. This is common in preclinical research, where negative findings are frequently underreported, potentially inflating pooled effect estimates (Dickersin, 1990; Sena et al., 2010). However, several considerations support the robustness of our core conclusions. First, the treatment effect direction was consistent across studies of varying sample sizes and precision, suggesting that the positive findings are not driven by a few outlier studies. Second, trim-and-fill analysis imputed zero missing studies for TST, FST, and OFT, and the pooled estimates remained unchanged, indicating that the observed asymmetry likely reflects true clinical or methodological heterogeneity rather than publication bias *per se*, but this finding should be interpreted cautiously due to the limited statistical power of detection methods in small-sample meta-analyses. Third, significant bias was not detected for cognitive (NOR) or some anxiety-like (EPM) outcomes, suggesting that artemisinin’s biological activity is not uniformly exaggerated across behavioral domains. Nevertheless, the exact magnitude of the pooled SMDs for depression-like outcomes may be somewhat inflated. We therefore caution against interpreting the effect sizes as precise efficacy estimates. Importantly, the directional consistency across outcomes suggests a true biological effect rather than an artifact of selective reporting, although the exact magnitude should be interpreted with caution. Prospective registration of animal studies and mandatory reporting of negative results are essential to mitigate this bias in future evidence syntheses (van der Worp et al., 2010; Macleod et al., 2014).

## 5 Limitations and directions for future research

This study presents the first systematic review and meta-analysis of preclinical evidence for the antidepressant effects of artemisinin and its derivatives. Based on the anti-inflammatory properties of artemisinin and existing animal experiments, we reposition artemisinin as a candidate for antidepressant drug development. Following the PICOS framework, a comprehensive search strategy was employed to minimize the risk of omitting relevant studies. Dispersed, small-sample animal study results were thus transformed into statistically robust estimates. Furthermore, not only depression-like behaviors, anxiety-like behaviors, and cognitive function were analyzed, but also the optimal therapeutic window for artemisinin in mood improvement was explored through subgroup analyses of intervention dose, frequency, duration, depression-like model type, rodent species, and derivative type.

This meta-analysis has several limitations. First, the small number of included studies and sample size (17 studies, 402 rodents) limit the precision of our findings. Second, all evidence derives from preclinical rodent models, and variability in species, depression-like models, derivatives, dosing regimens, and control groups introduces substantial clinical heterogeneity. Moreover, 94.1% of studies originated from a single country and predominantly used male rodents, markedly limiting external validity. Third, the included studies exhibited a high risk of bias, allocation concealment was inadequately reported in all 17 studies, and blinding was absent or unreported in most (14/17, 82.4%). These methodological deficiencies are established sources of systematic bias that can substantially inflate observed treatment effects. Under the GRADE framework, this high risk of bias, combined with substantial heterogeneity and indirectness, contributed to downgrading the certainty of evidence to very low for most outcomes. Therefore, the overall certainty of evidence derived from this body of literature is seriously compromised, and the antidepressant effects observed in these rodent models may not replicate under methodologically stringent conditions; the pooled effect sizes should be interpreted as potentially inflated estimates rather than precise quantifications of efficacy. Fourth, subgroup analyses were limited by small numbers of studies within individual subgroups, with several represented by only one or two studies. This severely restricted statistical power and the reliability of between-subgroup comparisons. Consequently, apparent differences in treatment effects across species, dosing regimens, or depression-like models may be spurious and should be treated as exploratory observations requiring confirmation in larger, more balanced datasets. We caution against using these findings to guide definitive decisions on experimental design or clinical translation until they are replicated in adequately powered studies. Finally, funnel plot asymmetry and significant Egger’s tests suggest potential publication bias. However, trim-and-fill analyses indicated no missing studies requiring imputation, supporting the robustness of the core conclusions. Nevertheless, the magnitude of the reported effect sizes should still be interpreted with caution given the limited number of studies. This limitation underscores the need for prospective registration of animal studies and mandatory reporting of negative results to improve the validity of future evidence syntheses.

## 6 Conclusion

This preclinical meta-analysis indicates that artemisinin and its derivatives exhibit significant antidepressant effects in rodent models of depression-like behavior, along with ancillary anxiolytic and cognitive-enhancing properties. These behavioral improvements are consistent with hypothesized mechanisms involving the inhibition of neuroinflammation and the enhancement of antioxidant defenses derived from individual preclinical studies, though the present meta-analysis was limited to behavioral endpoints and cannot establish these pathways directly. The magnitude of these effects was modulated by the method of depression-like induction, rodent species, and dosage regimen. However, the overall certainty of evidence is very low, primarily due to the high risk of bias across included studies—notably the universal lack of reported allocation concealment and inadequate blinding—coupled with substantial heterogeneity and the indirectness inherent to preclinical animal research. Although these findings provide preliminary support for clinical investigation of artemisinin-based therapies, 94.1% of studies originated from a single country and predominantly used male rodents, markedly limiting external validity and translatability to the female-predominant human population with depression.

## Data Availability

The original contributions presented in the study are included in the article/Supplementary Material, further inquiries can be directed to the corresponding author.
