# Supplementary material for: Antidepressant effects of artemisinin and its derivatives: a systematic review and meta-analysis of randomized controlled animal studies
Source: Front Pharmacol. 2026 Oct 5;17:1916381. doi: 10.3389/fphar.2026.1916381 (PMC13677903; doi:10.3389/fphar.2026.1916381)
Supplement: Supplementary file 1 [file Table1.docx]

**Table S1. Characteristics of the studies included in the meta-analysis.**

| **Author** | **Country** | **Year** | **Mouse strains** | **Mouse mode** | **Age（mean+SD）** | **Total/male/female** | **Weight（mean+SD）** | **Intervention** | **Control** | **Outcome** |
| --- | --- | --- | --- | --- | --- | --- | --- | --- | --- | --- |
| Zhuchunyan | China | 2018 | ICR mice | SNL | T+C:6-8 weeks old | T:12  C:12 | T+C: 26-28g | Intervention period：4-17days；Intervention frequency：once a day；Intervention dose： Dihydroartemisinin 40 mg/kg | Mouse model group | OFT; TST |
| Lei Wang | China | 2024 | C57BL/6J mice | CSDS | T+C:7-8 weeks old | T:10  C:10 | T+C: 22-24g | Intervention period：5days；Intervention frequency：once a day；Intervention dose：Artemisinin 10 mg/kg | Mouse model group | OFT; TST; FST |
| Lingling Li | China | 2024 | Sprague–Dawley rats | adult rats model of hypothyroidism | T+C:8 weeks old | T:10  C:10 | T+C: 22g | Intervention period：2 weeks；Intervention frequency：once a day；Intervention dose： Artemisinin 5 mg/kg | Mouse model group | TST; FST; EPM; MWM; Y-maze; NOR |
| Ruohong Lin | China | 2025 | C57BL/6 mice | CUMS | T+C:6-8 weeks old | T:8  C:8 | T+C: 18-21g | Intervention period：4 weeks；Intervention frequency： once a day； Intervention dose： Artemisinin 3 mg/kg | Mouse model group | TST; FST; SPT |
| Qing Liu | China | 2024 | rats | SPS | NA | T:20  C:20 | T+C: 20-25g | Intervention period：14 days；Intervention frequency： once a day；Intervention dose： Artemisinin 18 mg/kg | Mouse model group | OFT; EPM |
| Liuyutong | China | 2024 | C57BL/6J mice | CUMS | T+C：8 weeks old | T:10  C:10 | T+C：16-18g | Intervention period：28 days；Intervention frequency： once a day； Intervention dose： Dihydroartemisinin 50mg/kg | Mouse model group | OFT; TST; FST; SPT |
| Yejingjing | China | 2025 | C57BL/6 mice | CUMS | NA | T:12  C:12 | T+C：18-20g | Intervention period：4 weeks；Intervention frequency： once a day；Intervention dose：Artesunate 15mg/kg | Mouse model group | TST; FST; SPT |
| Yuting Gao | China | 2020 | C57BL6 mice | LPS | T+C：8 weeks old | T:10  C:10 | T+C：20-25g | Intervention period：4 days；Intervention frequency： once a day；Intervention dose：Dihydroartemisinin 40 mg/kg | Mouse model group | OFT; EPM; MWM |
| Chong Tang | China | 2024 | C57BL/6 mice | CUMS | T+C：6 weeks old | T:10  C:10 | T+C：20-25g | Intervention period：7 days；Intervention frequency： once a day；Intervention dose： Dihydroartemisinin 150 mg/kg | Mouse model group | OFT; TST; FST; SPT |
| Do Yeon Kim | Korea | 2023 | ICR mice | OVX | T+C：8 weeks old | T:9  C:9 | T+C：29-32g | Intervention period：8 weeks；Intervention frequency：once a day（Six times a week）；Intervention dose：Artemisia annua 300 mg/kg | Mouse model group | OFT; TST; EPM; Y-maze; NOR |
| Jingsi Liu | China | 2024 | C57BL/6 J mice | Anti-NMDAR Encephalitis Mouse Model | T+C：8-10 weeks old | T:25  C:25 | T+C：18-22g | Intervention period：14 days；Intervention frequency：twice a day； Intervention dose  ：Artesunate100 mg/kg | Mouse model group | OFT; EPM; NOR |
| Zhihao Liu | China | 2025 | C57BL/6J mice | CUMS | T+C：10 weeks old | T:8  C:8 | T+C：18-20g | Intervention period：3 weeks；Intervention frequency： once a day；Intervention dose  ：Absinthium L. essential oil 300 mg/kg | Mouse model group | OFT; FST; SPT |
| Shihao Huang | China | 2022 | ICR mice | LPS | T+C：7 weeks old | T:8  C:8 | T+C：NA | Intervention period：9 days；Intervention frequency： twice a day； Intervention dose： Artesunate 15 mg/kg | Mouse model group | OFT; TST; FST; SPT |
| Chunyan Zhu | China | 2022 | mice | SNL | NA | T:12  C:12 | NA | Intervention period：14 days；Intervention frequency： once a day； Intervention dose： Dihydroartemisinin：14.5 mg/kg | Mouse model group | TST; FST |
| Bailu | China | 2023 | rats | 3-MI Olfactory Dysfunction Model | T+C：6-8 weeks old | T:15  C:15 | T+C：20-22g | Intervention period：7 days；Intervention period： once a day； Intervention dose：Dihydroartemisinin 100 mg/kg | Mouse model group | OFT; EPM; SPT |
| Gaochao | China | 2024 | BALB /c mice | CUMS | T+C：8 weeks old | T:12  C:12 | T+C：18-20g | Intervention period：28 days；Intervention frequency： once a day； Intervention dose：Artemisinin ester 15 mg /kg | Mouse model group | FST; SPT |
| Liuying | China | 2023 | ICR mice | SNL | T+C： 8 weeks old | T:10  C:10 | T+C：30±2g | Intervention period：18 days；Intervention frequency：twice a day；Intervention dose：Dihydroartemisinin：16 mg/kg | Mouse model group | OFT; TST; FST; MWM |

*Note:* Spinal Nerve Ligation: SNL; Chronic Social Defeat Stress: CSDS; Chronic unpredictable mild stress: CUMS; Single Prolonged Stress: SPS; Lipopolysaccharide-induced depression model: LPS; Ovariectomy： OVX; 3-Methylindole: 3-MI; anti-N-methyl-D-aspartate receptor: Anti-NMDAR

Open Field Test: OFT; Tail Suspension Test: TST; Forced Swim Test: FST； Elevated Plus Maze Test: EPM; Morris Water Maze Test: MWM; Y-maze test: Y-maze; Novel Object Recognition Test: NOR; Sucrose Preference Test: SPT

**Table S2. Systematic Review Centre for Laboratory Animal Experimentation (SYRCLE).**

| Quality assessment | Selection bias |  |  | Performance bias |  | Detection bias |  |  |  |  |
| --- | --- | --- | --- | --- | --- | --- | --- | --- | --- | --- |
| References | Random sequences | Baseline characteristics | Allocation Concealment | Random housing | Blinding | Random outcome assessment | Blinding | Incomplete outcomes | Selecting report | Bias from |
|  | Generation |  |  |  |  |  |  | Data |  | other resources |
| Zhuchunyan，2018 | Low risk | Low risk | Unclear | Unclear | High risk | Unclear | High risk | Low risk | Low risk | Unclear |
| Lei Wang，2024 | Low risk | Low risk | Unclear | Unclear | Low risk | Unclear | Low risk | Low risk | Low risk | Low risk |
| Lingling Li，2024 | Low risk | Low risk | Unclear | Unclear | Unclear | Unclear | Unclear | Low risk | Low risk | Unclear |
| Ruohong Lin，2025 | Low risk | Low risk | Unclear | Unclear | Low risk | Unclear | Low risk | Low risk | Low risk | Low risk |
| Qing Liu，2024 | Low risk | Low risk | Unclear | Unclear | Unclear | Unclear | Low risk | Low risk | Low risk | Low risk |
| Liuyutong，2024 | Low risk | Low risk | Unclear | Unclear | Unclear | Unclear | Unclear | Low risk | Low risk | Low risk |
| Yejingjing，2025 | Low risk | Low risk | Unclear | Unclear | Unclear | Unclear | Unclear | Low risk | Low risk | Low risk |
| Yuting Gao，2020 | Low risk | Low risk | Unclear | Unclear | Unclear | Unclear | Unclear | Low risk | Low risk | Low risk |
| Chong Tang，2024 | Low risk | Low risk | Unclear | Unclear | Unclear | Unclear | Unclear | Low risk | Low risk | Low risk |
| Do Yeon Kim，2023 | Low risk | Low risk | Unclear | Unclear | Unclear | Unclear | Low risk | Low risk | Low risk | Low risk |
| Jingsi Liu，2024 | Low risk | Low risk | Unclear | Unclear | Low risk | Unclear | Low risk | Low risk | Low risk | Low risk |
| Zhihao Liu，2025 | Low risk | Low risk | Unclear | Unclear | Unclear | Unclear | Low risk | Low risk | Low risk | Low risk |
| Shihao Huang，2022 | Low risk | Low risk | Unclear | Unclear | Unclear | Unclear | Low risk | Low risk | Low risk | Low risk |
| Chunyan Zhu，2022 | Low risk | Low risk | Unclear | Unclear | Unclear | Unclear | Low risk | Low risk | Low risk | Low risk |
| Bailu，2023 | Low risk | Low risk | Unclear | Unclear | Unclear | Unclear | Low risk | Low risk | Low risk | Low risk |
| Gaochao，2024 | Low risk | Low risk | Unclear | Unclear | Unclear | Unclear | Low risk | Low risk | Low risk | Low risk |
| Liuying，2023 | Low risk | Low risk | Unclear | Unclear | Unclear | Unclear | Low risk | Low risk | Low risk | Low risk |

**Table S3.Subgroup analysis of Artemisinin, Dihydroartemisinin, and Artesunate.**

| OFT | subgroup | df | I2 | I2 P-val | Z | Z P-val | SMD | 95%CI | subgroup P-val |
| --- | --- | --- | --- | --- | --- | --- | --- | --- | --- |
|  |  | 2 |  |  |  |  |  |  | 0.0009 |
|  | Dihydroartemisinin | 5 | 84% | P < 0.001 | 4.93 | P < 0.001 | 3.67 | [2.21, 5.13] |  |
|  | Artesunate | 1 | 70% | 0.07 | 0.98 | 0.33 | 0.51 | [-0.52, 1.54] |  |
|  | Artemisinin | 1 | 0% | 0.88 | 3.18 | 0.001 | 0.86 | [0.33, 1.40] |  |
| TST | subgroup | df | I2 | I2 P-val | Z | Z P-val | SMD | 95%CI | subgroup P-val |
|  |  | 2 |  |  |  |  |  |  | 0.32 |
|  | Dihydroartemisinin | 4 | 83% | 0.0001 | 4.28 | P < 0.001 | -3.12 | [-4.54, -1.69] |  |
|  | Artesunate | 1 | 81% | 0.02 | 1.73 | 0.08 | -1.51 | [-3.22, 0.20] |  |
|  | Artemisinin | 2 | 86% | 0.0006 | 2.01 | 0.04 | -1.89 | [-3.73, -0.04] |  |
| FST | subgroup | df | I2 | I2 P-val | Z | Z P-val | SMD | 95%CI | subgroup P-val |
|  |  | 2 |  |  |  |  |  |  | 0.32 |
|  | Dihydroartemisinin | 3 | 84% | 0.0003 | 3.59 | 0.0003 | -3.02 | [-4.67, -1.37] |  |
|  | Artesunate | 2 | 88% | 0.0003 | 3.25 | 0.001 | -5.19 | [-8.32, -2.06] |  |
|  | Artemisinin | 2 | 86% | 0.0008 | 2.27 | 0.02 | -2.32 | [-4.32, -0.32] |  |
| EPM | subgroup | df | I2 | I2 P-val | Z | Z P-val | SMD | 95%CI | subgroup P-val |
|  |  | 2 |  |  |  |  |  |  | 0.33 |
|  | Dihydroartemisinin | 1 | 87% | 0.005 | 1.77 | 0.08 | -2.05 | [-4.32, 0.22] |  |
|  | Artesunate |  |  |  | 2.52 | 0.01 | -0.74 | [-1.31, -0.16] |  |
|  | Artemisinin | 1 | 90% | 0.002 | 1.63 | 0.1 | -2.17 | [-4.77, 0.43] |  |
| MWM | subgroup | df | I2 | I2 P-val | Z | Z P-val | SMD | 95%CI | subgroup P-val |
|  |  | 1 |  |  |  |  |  |  | 0.04 |
|  | Dihydroartemisinin | 1 | 94% | P < 0.001 | 1.34 | 0.18 | -3.88 | [–9.54, 1.79] |  |
|  | Artemisinin | NA | NA | NA | 5.28 | P < 0.001 | -6.5 | [-8.91, -4.09] |  |
| NOR | subgroup | df | I2 | I2 P-val | Z | Z P-val | SMD | 95%CI | subgroup P-val |
|  |  | 1 |  |  |  |  |  |  | 0.86 |
|  | Artesunate | NA | NA | NA | 5.56 | P < 0.001 | 0.25 | [0.16, 0.34] |  |
|  | Artemisinin | NA | NA | NA | 6.66 | P < 0.001 | 0.24 | [0.17, 0.31] |  |
| SPT | subgroup | df | I2 | I2 P-val | Z | Z P-val | SMD | 95%CI | subgroup P-val |
|  |  | 2 |  |  |  |  |  |  | 0.2 |
|  | Dihydroartemisinin | 2 | 48% | 0.15 | 3.8 | 0.0001 | 1.51 | [0.73, 2.29] |  |
|  | Artesunate | 2 | 91% | P < 0.001 | 2.68 | 0.007 | 4.31 | [1.16, 7.46] |  |
|  | Artemisinin | NA | NA | NA | 3.2 | 0.001 | 2.11 | [0.82, 3.41] |  |

**Note:Artemisia annua extract (Do Yeon Kim, 2023) and absinthium L. essential oil (Zhihao Liu, 2025) were excluded from the derivative-type subgroup. The study by Gaochao et al. (2024) using artemisinin ester was classified under artesunate,**

**Table S4. Grading of Recommendations Assessment, Development and Evaluation(GRADE).**

| Outcome | Studies (n) | Sample Size (approx.) | Effect Estimate \[95% CI] | Risk of Bias | Inconsistency | Indirectness | Imprecision | Publication Bias | Certainty of Evidence |
| --- | --- | --- | --- | --- | --- | --- | --- | --- | --- |
| Primary Outcomes: Depression-like Behaviors |  |  |  |  |  |  |  |  |  |
| TST immobility time | 11 | 240 | SMD −2.32 \[−3.14, −1.49] | Serious −1 | Serious −1 | Serious −1 | Not serious | Serious −1 | ⊕○○○ Very low |
| FST immobility time | 11 | 240 | SMD −3.21 \[−4.25, −2.17] | Serious −1 | Serious −1 | Serious −1 | Not serious | Serious −1 | ⊕○○○ Very low |
| SPT sucrose preference | 8 | 180 | SMD 2.45 \[1.45, 3.45] | Serious −1 | Serious −1 | Serious −1 | Not serious | Not serious | ⊕○○○ Very low |
| Secondary Outcomes: Anxiety-like Behaviors |  |  |  |  |  |  |  |  |  |
| OFT central zone time | 12 | 280 | SMD 2.04 \[1.27, 2.80] | Serious −1 | Serious −1 | Serious −1 | Not serious | Serious −1 | ⊕○○○ Very low |
| EPM closed-arm time | 6 | 140 | SMD −1.59 \[−2.35, −0.83] | Serious −1 | Serious −1 | Serious −1 | Serious −1 | Not serious | ⊕○○○ Very low |
| Secondary Outcomes: Cognitive Function |  |  |  |  |  |  |  |  |  |
| MWM escape latency | 3 | 70 | SMD −4.72 \[−9.04, −0.39] | Serious −1 | Serious −1 | Serious −1 | Serious −1 | Not serious | ⊕○○○ Very low |
| Y-maze novel-arm latency | 2 | 50 | SMD −2.60 \[−4.69, −0.50] | Serious −1 | Serious −1 | Serious −1 | Serious −1 | Not serious | ⊕○○○ Very low |
| NOR discrimination index | 3 | 70 | SMD 2.13 \[1.26, 3.00] | Serious −1 | Not serious | Serious −1 | Serious −1 | Not serious | ⊕⊕○○ Low |

**Note: The initial certainty of evidence was rated as high (⊕⊕⊕⊕) based on the inclusion of randomized controlled trials (RCTs), and subsequently downgraded according to the five GRADE domains.**

**Table S5.Trim-and-Fill analysis.**

| Measure | Random-effects Estimate | 95% CI | Q (p) | τ² | Studies to Trim | Change |
| --- | --- | --- | --- | --- | --- | --- |
| **OFT** | **2.036** | **1.274 ~ 2.798** | 70.955 (<0.001) | **1.411** | 0 | No change |
| **TST** | **−2.319** | **−3.144 ~ −1.494** | 52.085 (<0.001) | **1.508** | 0 | No change |
| **FST** | **−3.210** | **−4.249 ~ −2.171** | 61.493 (<0.001) | **2.438** | 0 | No change |

**Table S6. Rationale for Study Selection.**

| Artemisinin neurotoxicity: Neuropathology in rats and mechanistic studies in vitro | S. Kamchonwongpaisan | Lack of quantitative efficacy assessment data. |
| --- | --- | --- |
| The effect of 10α-trifluoromethylhydroartemisinin on Plasmodium berghei infection and its toxicity in experimental animals | P. D. Binh | Lack of quantitative data related to neuroprotection. |
| Artemisinin inhibits inducible nitric oxide synthase and nuclear factor NF-kB activation | E. Aldieri | Incomplete data reporting. |
| Chronic administration of Artemisia annua L. attenuates oxidative stress induced by D-galactose in mice | M. H. Kim | Incomplete data reporting. |
| Powerful antioxidant and pro-oxidant properties of Cassia roxburghii DC. leaves cultivated in Egypt in relation to their anti-infectious activities | S. S. M. El Souda | Lack of original experimental data on artemisinin's neuroprotective effects. |
| Hippocampal PPARδ overexpression or activation represses stress-induced depressive behaviors and enhances neurogenesis | M. J. Ji | Lack of original experimental data on artemisinin's neuroprotective effects. |
| Antimalarial Drug Artemether Inhibits Neuroinflammation in BV2 Microglia Through Nrf2-Dependent Mechanisms | U. P. Okorji | Lack of in vivo quantitative efficacy data and complete statistical information. |
| Neuroprotective effects in PC12 cells by artemether, a clinical antimalarial agent | L. Zhang | Lack of in vivo quantitative efficacy data and complete statistical information. |
| Artemisinin conferred ERK mediated neuroprotection to PC12 cells and cortical neurons exposed to sodium nitroprusside-induced oxidative insult | W. Zheng | Lack of in vivo quantitative efficacy data and complete statistical information. |
| Neuroprotective effects of artemisinin against isoflurane-induced cognitive impairments and neuronal cell death involve JNK/ERK1/2 signalling and improved hippocampal histone acetylation in neonatal rats | G. Xu | Incomplete data reporting. |
| Incorporation of Dihydroartemisinin into Memantine Through a Propriate Spacer to Make Hybrid with Enhanced Effects to Protect PC12 Cells from Corticosterone-caused Impairments | L. Zhang | Lack of standardized efficacy assessment data. |
| Artemisinin conferred ERK-mediated neuroprotection to PC12 cells and cortical neurons exposed to sodium nitroprussideinduced oxidative insult | W. Zheng | Lack of in vivo quantitative efficacy data and complete statistical information. |
| Artemisinin prevents glutamate-induced neuronal cell death via Akt pathway activation | S. P. Lin | Incomplete data reporting. |
| Artesunate suppresses oxidative and inflammatory processes by activating Nrf2 and ROS-dependent p38 MAPK and protects against cerebral ischemia-reperfusion injury | H. Lu | Incomplete data reporting. |
| The protective effect of artemisinin against oxidative stress in neuronal/non neuronal cells and its underlying mechanisms | X. Xigan | Incomplete data reporting. |
| Elucidating the Molecular Basis for Inhibitory Neurotransmission Regulation by Artemisinins | V. B. Kasaragod | Lack of experimentally validated quantitative efficacy data. |
| Structure of Heteropentameric GABAA Receptors and Receptor-Anchoring Properties of Gephyrin | V. B. Kasaragod | Lack of quantitative efficacy assessment data. |
| Targeting GABAAR-Associated proteins: new modulators, labels and concepts | V. Khayenko | Lack of original experimental data on artemisinin's neuroprotective effects. |
| Bridges between Antimalarials and Synaptic Transmission | J. J. Kim | Unable to extract effect size. |
| Neuronal maturation in the hippocampal dentate gyrus via chronic oral administration of Artemisa annua extract is independent of cyclooxygenase 2 signaling pathway in diet-induced obesity mouse model | P. S. Kim | Incomplete data reporting. |
| More than anti-malarial agents: Therapeutic potential of artemisinins in neurodegeneration | B. W. Lu | Only figures without specific numerical values. |
| Design, Synthesis, and Mechanism of Dihydroartemisinin⁻Coumarin Hybrids as Potential Anti-Neuroinflammatory Agents | H. Yu | Lack of standardized efficacy assessment data. |
| Artemisinin attenuated hydrogen peroxide (H2o2)-induced oxidative injury in SH-SY5Y and hippocampal neurons via the activation of AMPK pathway | X. Zhao | Lack of in vivo quantitative efficacy data and complete statistical information. |
| Pyridoxal kinase inhibition by artemisinins down-regulates inhibitory neurotransmission | V. B. Kasaragod | Lack of quantitative efficacy assessment data. |
| Artemisinin protects motoneurons against axotomy-induced apoptosis through activation of the PKA-Akt signaling pathway and promotes neural stem/progenitor cells differentiation into NeuN+ neurons | L. Liu | Incomplete data reporting. |
| Precisely embedding active sites into a mesoporous Zr-framework through linker installation for high-efficiency photocatalysis | J. Pang | Lack of standardized efficacy assessment data. |
| Dihydroartemisinin ameliorates cyclophosphamide-induced chronic cystitis via the suppression of NF-jB pathway and pyroptosis | J. Wang | Lack of quantitative data related to neuroprotection. |
| Artesunate inhibits the mevalonate pathway and promotes glioma cell senescence | S. Wei | Lack of quantitative data related to neuroprotection. |
| Neuroinflammation in CNS diseases: Molecular mechanisms and the therapeutic potential of plant derived bioactive molecules | M. O. Welcome | Lack of quantitative efficacy assessment data. |
| Artesunate promotes the proliferation of neural stem/progenitor cells and alleviates Ischemia-reperfusion Injury through PI3K/Akt/FOXO-3a/p27kip1 signaling pathway | K. Zhang | Incomplete data reporting. |
| Artemisinin improved neuronal functions in Alzheimer's disease animal model 3xtg mice and neuronal cells via stimulating the ERK/CREB signaling pathway | X. Zhao | Only figures without specific numerical values. |
| Dihydroartemisinin Ameliorates Learning and Memory in Alzheimer’s Disease Through Promoting Autophagosome-Lysosome Fusion and Autolysosomal Degradation for Aβ Clearance | Y. Zhao | Incomplete data reporting. |
| Artesunate attenuates traumatic brain injuryinduced impairments in rats | Z. Zhou | Incomplete data reporting. |
| Evaluation of the therapeutic potential of dihydroartemisinin and its dimer as b-amyloid aggregation and b-secretase inhibitors | Y. Hambardzumyan | Lack of overall neuroprotective efficacy data. |
| Synergistic integration of dihydro-artemisinin with γ-aminobutyric acid results in a more potential anti-depressant | Y. He | Incomplete data reporting. |
| The NLRP3 inflammasome: A potential therapeutic target for traumatic brain injury | S. Ismael | Unable to extract effect size. |
| Artesunate restores the levels of inhibitory synapse proteins and reduces amyloid-β and C-terminal fragments (CTFs) of the amyloid precursor protein in an AD-mouse model | E. Kiss | Incomplete data reporting. |
| Artemether confers neuroprotection on cerebral ischemic injury through stimulation of the Erk1/2-P90rsk-CREB signaling pathway | S. Li | Incomplete data reporting. |
| Artesunate attenuates inflammatory injury and inhibits the NF-κB pathway in a mouse model of cerebral ischemia | Y. Liu | Only figures without specific numerical values. |
| Cannabidiol-dihydroartemisinin conjugates for ameliorating neuroinflammation with reduced cytotoxicity | F. Wang | Lack of standardized efficacy assessment data. |
| Traditional Chinese Medicine Monomers: Novel Strategy for Endogenous Neural Stem Cells Activation After Stroke | J. Wang | Unable to extract effect size. |
| Autophagy in vascular dementia and natural products with autophagy regulating activity | W. Wang | Unable to extract effect size. |
| Dihydroartemisinin Induces O-GlcNAcylation and Improves Cognitive Function in a Mouse Model of Tauopathy | L. Xia | Incomplete data reporting. |
| Artemisinin inhibits TLR4 signaling by targeting co-receptor MD2 in microglial BV-2 cells and prevents lipopolysaccharide-induced blood–brain barrier leakage in mice | T. Zhang | Lack of quantitative efficacy assessment data. |
| Artemisinin Alleviates Cerebral Ischemia/Reperfusion-Induced Oxidative Damage via Regulating PHB2-Mediated Autophagy in the Human Neuroblastoma SH-SY5Y Cell Line | M. Jiang | Lack of in vivo quantitative efficacy data and complete statistical information. |
| Artemisinin-treatment in pre-symptomatic APP-PS1 mice increases gephyrin phosphorylation at Ser270: A modification regulating postsynaptic GABAAR density | E. Kiss | Incomplete data reporting. |
| Current Progress on Neuroprotection Induced by Artemisia, Ginseng, Astragalus, and Ginkgo Traditional Chinese Medicines for the Therapy of Alzheimer's Disease | Q. Li | Unable to extract effect size. |
| The emerging roles of ferroptosis in cells of the central nervous system | Y. Li | Unable to extract effect size. |
| Drug discovery from natural products – Old problems and novel solutions for the treatment of neurodegenerative diseases | U. Maitra | Unable to extract effect size. |
| Dihydroartemisinin attenuates hypoxic-ischemic brain damage in neonatal rats by inhibiting oxidative stress | Q. Xiong | Incomplete data reporting. |
| Neurogenesis and Proliferation of Neural Stem/Progenitor Cells Conferred by Artesunate via FOXO3a/p27Kip1 Axis in Mouse Stroke Model | K. Zhang | Only figures without specific numerical values. |
| Dihydroartemisinin ameliorates cerebral I/R injury in rats via regulating VWF and autophagy-mediated SIRT1/FOXO1 pathway | Q. Duan | Only figures without specific numerical values. |
| Efficacy of Artemisia annua Linné in improving cognitive impairment in a chronic cerebral hypoperfusion-induced vascular dementia animal model | S. Y. Kim | Incomplete data reporting. |
| Anti-malaria drug artesunate prevents development of amyloid-β pathology in mice by upregulating PICALM at the blood-brain barrier | K. Kisler | Unable to extract effect size. |
| Effects of Artemisinin on Anti-epileptogenic, Antioxidant and Cholinesterase Enzymes in Pentylenetetrazole-induced Kindling Model in Mice | Y. Kocak | Incomplete data reporting. |
| Protective Effect of Artemisinin on Cerebral Ischemic Stroke Via PI3k/Akt/GSK-3β Signaling Pathway Activation | R. Li | Incomplete data reporting. |
| Artesunate alleviates intracerebral haemorrhage secondary injury by inducing ferroptosis in M1-polarized microglia and suppressing inflammation through AMPK/mTORC1/GPX4 pathway | G. Xie | Incomplete data reporting. |
| PKM2 Nuclear Translocation Promotes Glial Cell Activation and Aggravates the Brain Injury of Intracerebral Hemorrhage | X. Y. Xiong | Lack of original experimental data on artemisinin's neuroprotective effects. |
| Scopoletin as Key Component of a Novel Acaricidal Agent and Its Production Methodology | G. Chen | Lack of original experimental data on artemisinin's neuroprotective effects. |
| Pre-clinical studies comparing the anti-inflammatory potential of artemisinic compounds by targeting NFκB/TNF-α/NLRP3 and Nrf2/TRX pathways in Balb/C mice | S. T. B. Kazmi | Incomplete data reporting. |
| Another Use for a Proven Drug: Experimental Evidence for the Potential of Artemisinin and Its Derivatives to Treat Alzheimer’s Disease | E. Kiss | Only figures without specific numerical values. |
| Artemisinin ameliorates thyroid function and complications in adult male hypothyroid rats via upregulation of the L1 cell adhesion molecule | L. L. Li | Lack of quantitative data related to neuroprotection. |
| Artemisinin protects dopaminergic neurons against 1-methyl-4-phenyl-1,2,3,6-tetrahydropyridine-induced neurotoxicity in a mouse model of Parkinson's disease | H. S. Lim | Lack of quantitative assessment data for neuroprotective efficacy. |
| Exploring the neuroprotective role of artesunate in mouse models of anti-NMDAR encephalitis: insights from molecular mechanisms and transmission electron microscopy | J. Liu | Lack of quantitative efficacy assessment data. |
| Interventional effect and inflammatory regulation mechanism of dihydroartemisinin combined with pregabalin on neuropathic pain mice | Y. Liu | Unable to extract net effect data of artemisinin alone. |
| Xylopia parviflora (A. rich.) benth. Mitigates anxiety behavior and chronic mild stress-induced depression-like behavior in mice: The involvement of biogenic amine neurotransmitters, cyclooxygenase-2 and stress biomarkers in its antidepressant activity | A. A. Murtala | Lack of original experimental data on artemisinin's neuroprotective effects. |
| Artemisinin attenuated ischemic stroke induced pyroptosis by inhibiting ROS/TXNIP/NLRP3/Caspase-1 signaling pathway | Y. Wang | Incomplete data reporting. |
| Ferroptosis: A New Research Direction of Artemisinin and Its Derivatives in Anti-Cancer Treatment | Y. Wang | Unable to extract effect size. |
| Targeting m6A mRNA demethylase FTO alleviates manganese-induced cognitive memory deficits in mice | Y. Wen | Not artemisinin compounds; no original data on artemisinin neuroprotection. |
| Dihydroartemisinin promotes tau O-GlcNAcylation and improves cognitive function in hTau transgenic mice | L. Xia | Incomplete data reporting. |
| The Potential Role of Artemisinins Against Neurodegenerative Diseases | L. Xia | Unable to extract effect size. |
| Transcriptome and metabolome analysis reveals PRV XJ delgE/gI/TK protects intracranially infected mice from death by regulating the inflammation | L. Xu | Lack of original experimental data on artemisinin's neuroprotective effects. |
| Elucidation of Artemisinin as a Potent GSK3β Inhibitor for Neurodegenerative Disorders via Machine Learning-Driven QSAR and Virtual Screening of Natural Compounds | H. H. Alhassan | Lack of experimentally validated quantitative efficacy data. |
| Artemisinin Ameliorates the Neurotoxic Effect of 3-Nitropropionic Acid: A Possible Involvement of the ERK/BDNF/Nrf2/HO-1 Signaling Pathway | R. Arthur | Incomplete data reporting. |
| Treament with dihydroartemisinin improves response of normoxic and hypoxic lung cancer cells to radiation therapy | S. Bader | Lack of quantitative data related to neuroprotection. |
| Artemether Improves Aβ1-42-Induced Mitochondrial Dysfunction and Protects Against Blood–Brain Barrier Damage Through Activating the CAMKK2/AMPK/PGC1α Signaling Pathway | J. Cai | Incomplete data reporting. |
| Artemisinin alleviates astrocyte overactivation and neuroinflammation by modulating the IRE1/NF-κB signaling pathway in in vitro and in vivo Alzheimer's disease models | L. Chen | Only figures without specific numerical values. |
| Artemisinin antagonizes cognitive disorder in hyperuricemia through glutamate receptor-interacting protein 1-suppressed pyroptosis | Y. Chen | Only figures without specific numerical values. |
| Artesunate alleviates Parkinson's disease by targeting astrocyte MT2A to attenuate dopamine neuronal cuproptosis | Y. Cheng | Incomplete data reporting. |
| Protective effects of artesunate on the neurovascular unit by regulating PDGFRβ signaling in experimental cerebral malaria | Y. Dai | Lack of quantitative data related to neuroprotection. |
| Artemisinin inhibits neuronal ferroptosis in Alzheimer’s disease models by targeting KEAP1 | P. X. Deng | Incomplete data reporting. |
| Therapeutic effect of dihydroartemisinin on Alzheimer’s disease model mice with senile macular degeneration | G. Han | Incomplete data reporting. |
| Artesunate attenuates cerebral injury after cardiac arrest via inhibiting caspase-11-mediated pyroptosis through NEDD4 | L. He | Only figures without specific numerical values. |
| Neuronal protective effect of Artemisinin in ischemic stroke: Achieved by blocking lysine demethylase 1A-mediated demethylation of sphingosine kinase 2 | H. Li | Incomplete data reporting. |
| Artemisinin alleviates ischemic stroke injury and promotes neurogenesis through PPARγ-mediated M2 polarization of microglia | L. Li | Incomplete data reporting. |
| Nilotinib attenuates vascular pathology in experimental cerebral malaria | L. S. Ortolan | Lack of original experimental data on artemisinin's neuroprotective effects. |
| Polycyclitol Derivatives Restore Long- Term Memory Via cdk5/p25 Activation of Tau Signaling in Experimental Cerebral Malaria | P. K. Simhadri | Lack of original experimental data on artemisinin's neuroprotective effects. |
| Artesunate alleviated hippocampal neuron pyroptosis by down-regulating NLRP3 in rats with cerebral small vessel disease | X. Wang | Incomplete data reporting. |
| Unlocking the neuroprotective secrets of natural products: a focus on the gut-brain axis | X. Yang | Unable to extract effect size. |
| Artemisinin alleviates Parkinson’s disease by targeting Adcy5-Gch1 axis to trigger a cascade generation of BH4 and dopamine in rats | X. X. Yang | Incomplete data reporting. |
| Luteolin ameliorates chronic stress-induced depressive-like behaviors in mice by promoting the Arginase-1+ microglial phenotype via a PPARγ-dependent mechanism | N. J. Yuan | Lack of original experimental data on artemisinin's neuroprotective effects. |
| KLHL8-mediated ubiquitination and TAX1BP1-dependent autophagic degradation of GPX4 drive neuronal ferroptosis | J. Zhao | Lack of original experimental data on artemisinin's neuroprotective effects. |
| Regeneration of Phytochemicals by Structure-Driven Organization of Microbial Biosynthetic Steps | Xue Ming Wu | Lack of quantitative data for efficacy assessment. |
| A mitochondria-targeting artemisinin derivative with sharply increased antitumor but depressed anti-yeast and anti-malaria activities | Chen Sun | Lack of standardized efficacy assessment data. |
| Inhibition of metalloproteinase-9 secretion and gene expression by artemisinin derivatives | Daniele Magenta | Incomplete data reporting. |
| Enhanced antidepressant effects of fluoxetine combined with quercetin: studies in vitro and in vivo | Ran Yang | Lack of original experimental data on artemisinin's neuroprotective effects. |
| Reversible elimination of K+ and Na+ currents at motor nerve terminals by SM486, a derivative of artemisinin | Y L Shi | Lack of standardized neuroprotective efficacy data. |
| Polystyrene microplastics induce anxiety via HRAS derived PERK-NF-κB pathway | Guanjun Li | Lack of original experimental data on artemisinin's neuroprotective effects. |
| An efficient partial synthesis of (+)-artemisinin and (+)-deoxoartemisinin | P. T. Lansbury | Lack of standardized efficacy assessment data. |
| Identification and quantification of sesquiterpenes, alkaloids, flavonoids and coumarins in Artemisia annua L: Potential artemisinin activity-enhancing compounds | J. Zhang | Lack of pharmacodynamic data. |
| Artemisinin-hydroxychloroquine combination from traditional antimalarial medicine attenuates pulmonary fibrosis via PI3K/AKT/GSK3β-mediated inhibition of epithelial-mesenchymal transition | L. Zhang | Lack of quantitative data related to neuroprotection. |
| Chemical constituents from Artemisia annua with potential anti-inflammatory activities | Q. G. Wu | Lack of standardized efficacy assessment data. |
| Integrative lipophilicity assessment and pharmacokinetic correlation of pyrimidine precursors and artesunate–pyrimidine hybrids: development of QSAR models for anticancer activity and interaction with P-glycoprotein | M. Mosić | Lack of experimentally validated quantitative efficacy data. |
| Artesunate attenuates pulmonary fibrosis by suppressing fibroblast senescence through inhibition of the STAT3/p53 signaling pathway | J. Ding | Lack of quantitative data related to neuroprotection. |
| Targeted inhibition of colorectal cancer by nano-dihydroartemisinin liposomes: Insights into the disruption of ABCG2 and BCL2 leading to induction of oxidative stress and metabolic disorder | J. F. Cao | Lack of standardized efficacy assessment data. |
| Dihydroartemisinin inhibits the development of autoimmune thyroiditis by modulating oxidative stress and immune imbalance | S. Zhu | Lack of quantitative data related to neuroprotection. |
| Artesunate overcomes icotinib resistance in non-small cell lung cancer with EGFR -sensitive mutations | T. Zhao | Lack of standardized efficacy assessment data. |
| Engineered macrophage membrane-coated dihydroartemisinin nanoparticles with enhanced CCR2 expression improved symptoms in MRL/LPR mice by metabolic reprogramming of proinflammatory macrophages | L. Zhao | Lack of quantitative data related to neuroprotection. |
| JAK3/STAT5A-dependent IL-8 regulation drives ESCC angiogenesis and is suppressed by dihydroartemisinin | K. Zhang | Lack of standardized efficacy assessment data. |
| Study on a TCM evaluation method based on an MIP-modified MOF sensor with highly selective electrocatalytic activity-an Artemisia annua L. perspective | J. Zhang | Lack of quantitative data related to neuroprotection. |
| Dihydroartemisinin ameliorates hemarthrosis-induced cartilage degeneration by suppressing chondrocyte senescence via activation of Keap1-Nrf2 signaling pathway | Q. Zeng | Lack of quantitative data for efficacy assessment. |
| Dihydroartemisinin ameliorates cartilage degeneration in hemophilic arthropathy by suppressing chondrocyte senescence via activation of Nrf2 signaling pathway | Q. Zeng | Lack of quantitative data related to neuroprotection. |
| Metal-phenolic network crosslinked nanogel with prolonged biofilm retention for dihydroartemisinin/NIR synergistically enhanced chemodynamic therapy | W. Yu | Lack of quantitative data related to neuroprotection. |
| Artesunate carbon dots for attenuating periodontal inflammation and promoting bone regeneration via activation of AMPK | S. Yu | Lack of standardized efficacy assessment data. |
| Acevaltrate as a novel ferroptosis inducer with dual targets of PCBP1/2 and GPX4 in colorectal cancer | D. Yu | Lack of standardized efficacy assessment data. |

**Table S7. Search Strategy.**

| **PUBMED** |  |
| --- | --- |
| #7 | ((((((Depression[MeSH Terms]) OR (Depressive Disorder[MeSH Terms])) OR (Depression, Chemical[MeSH Terms])) OR (Depressive Disorder, Treatment-Resistant[MeSH Terms])) OR (Major Depressive Disorder[MeSH Terms])) OR ((((((((((((((((((((((((((((((((((((((((((((((((((((((((((((((((((((((((Depression[Title/Abstract]) OR (Depressive Disorder[Title/Abstract])) OR (Depression, Chemical[Title/Abstract])) OR (Depressive Disorder, Treatment-Resistant[Title/Abstract])) OR (Major Depressive Disorder[Title/Abstract])) OR (Depressive Symptoms[Title/Abstract])) OR (Depressive Symptom[Title/Abstract])) OR (Symptom, Depressive[Title/Abstract])) OR (Emotional Depression[Title/Abstract])) OR (Depression, Emotional[Title/Abstract])) OR (Depressive Disorders[Title/Abstract])) OR (Disorder, Depressive[Title/Abstract])) OR (Disorders, Depressive[Title/Abstract])) OR (Depression Disorder[Title/Abstract])) OR (Disorders, Depression[Title/Abstract])) OR (Neurosis, Depressive[Title/Abstract])) OR (Depressive Neuroses[Title/Abstract])) OR (Neuroses, Depressive[Title/Abstract])) OR (Depression, Endogenous[Title/Abstract])) OR (Depressions, Endogenous[Title/Abstract])) OR (Endogenous Depression[Title/Abstract])) OR (Melancholia[Title/Abstract])) OR (Unipolar Depression[Title/Abstract])) OR (Depression, Unipolar[Title/Abstract])) OR (Depressive Syndrome[Title/Abstract])) OR (Syndrome, Depressive[Title/Abstract])) OR (Syndromes, Depressive[Title/Abstract])) OR (Depression, Neurotic[Title/Abstract])) OR (Depressions, Neurotic[Title/Abstract])) OR (Neurotic Depression[Title/Abstract])) OR (Chemical Depression[Title/Abstract])) OR (Chemical Depressions[Title/Abstract])) OR (Depressions, Chemical[Title/Abstract])) OR (Depressive Disorders, Treatment-Resistant[Title/Abstract])) OR (Depressive Disorder, Treatment Resistant[Title/Abstract])) OR (Disorders, Treatment-Resistant Depressive[Title/Abstract])) OR (Disorder, Treatment-Resistant Depressive[Title/Abstract])) OR (Treatment-Resistant Depressive Disorder[Title/Abstract])) OR (Treatment-Resistant Depressive Disorders[Title/Abstract])) OR (Treatment Resistant Depression[Title/Abstract])) OR (Depressions, Treatment Resistant[Title/Abstract])) OR (Depression, Treatment Resistant[Title/Abstract])) OR (Resistant Depressions, Treatment[Title/Abstract])) OR (Resistant Depression, Treatment[Title/Abstract])) OR (Treatment Resistant Depressions[Title/Abstract])) OR (Refractory Depression[Title/Abstract])) OR (Depression, Refractory[Title/Abstract])) OR (Depressions, Refractory[Title/Abstract])) OR (Refractory Depressions[Title/Abstract])) OR (Therapy-Resistant Depression[Title/Abstract])) OR (Depressions, Therapy-Resistant[Title/Abstract])) OR (Depression, Therapy-Resistant[Title/Abstract])) OR (Therapy Resistant Depression[Title/Abstract])) OR (Therapy-Resistant Depressions[Title/Abstract])) OR (Depressive Disorders, Major[Title/Abstract])) OR (Disorder, Major Depressive[Title/Abstract])) OR (Disorders, Major Depressive[Title/Abstract])) OR (Major Depressive Disorders[Title/Abstract])) OR (Clinical Depression[Title/Abstract])) OR (Depressions, Clinical[Title/Abstract])) OR (Major Depression Disorder[Title/Abstract])) OR (Depression Disorders, Major[Title/Abstract])) OR (Disorders, Major Depression[Title/Abstract])) OR (Depressive Disorder, Major[Title/Abstract])) OR (Depression, Involutional[Title/Abstract])) OR (Depressions, Involutional[Title/Abstract])) OR (Melancholia, Involutional[Title/Abstract])) OR (Melancholias, Involutional[Title/Abstract])) OR (Psychosis, Involutional[Title/Abstract])) OR (Psychoses, Involutional[Title/Abstract])) OR (Paraphrenia, Involutional[Title/Abstract])) OR (Paraphrenias, Involutional[Title/Abstract]))) AND (((Artemisinins[MeSH Terms]) OR (artemisinin[MeSH Terms])) OR (((((((((((((((((((((((((((((((Artemisinins[Title/Abstract]) OR (artemisinin[Title/Abstract])) OR (arteannuin[Title/Abstract])) OR (qinghaosu[Title/Abstract])) OR (quing hau sau[Title/Abstract])) OR (quinghaosu[Title/Abstract])) OR (qing hao su[Title/Abstract])) OR (artemisininum[Title/Abstract])) OR (Artemisinine[Title/Abstract])) OR (artemisinin-naphthoquine combination[Title/Abstract])) OR (artemisinin G[Title/Abstract])) OR (artenimol[Title/Abstract])) OR (10-(2-(3-trifluoromethylphenylpiperazyl)ethyl)deoxoartemisinin[Title/Abstract])) OR (dihydroartemisinin beta-glucuronide[Title/Abstract])) OR (10-(4-trifluoromethylphenoxy)dihydroartemisinin[Title/Abstract])) OR (artemisinin trioxane alcohol[Title/Abstract])) OR (anhydrodihydroartemisinin[Title/Abstract])) OR (artemisinin B[Title/Abstract])) OR (deoxoartemisinin[Title/Abstract])) OR (Artemisia annua[Title/Abstract])) OR (sulfadoxine-pyrimethamine-artesunate[Title/Abstract])) OR (artemisinic aldehyde delta11(13) reductase, Artemisia annua[Title/Abstract])) OR (ethyl 2-(4-(12-beta-artemisininoxy))phenoxylpropionate[Title/Abstract])) OR (SM905[Title/Abstract])) OR (amorpha-4,11-diene[Title/Abstract])) OR (Artemisone[Title/Abstract])) OR (99-357 compound[Title/Abstract])) OR (SM 486[Title/Abstract])) OR (Artemether[Title/Abstract])) OR (Artesunate[Title/Abstract])) OR (artesumycin[Title/Abstract]))) |
| #6 | ((Artemisinins[MeSH Terms]) OR (artemisinin[MeSH Terms])) OR (((((((((((((((((((((((((((((((Artemisinins[Title/Abstract]) OR (artemisinin[Title/Abstract])) OR (arteannuin[Title/Abstract])) OR (qinghaosu[Title/Abstract])) OR (quing hau sau[Title/Abstract])) OR (quinghaosu[Title/Abstract])) OR (qing hao su[Title/Abstract])) OR (artemisininum[Title/Abstract])) OR (Artemisinine[Title/Abstract])) OR (artemisinin-naphthoquine combination[Title/Abstract])) OR (artemisinin G[Title/Abstract])) OR (artenimol[Title/Abstract])) OR (10-(2-(3-trifluoromethylphenylpiperazyl)ethyl)deoxoartemisinin[Title/Abstract])) OR (dihydroartemisinin beta-glucuronide[Title/Abstract])) OR (10-(4-trifluoromethylphenoxy)dihydroartemisinin[Title/Abstract])) OR (artemisinin trioxane alcohol[Title/Abstract])) OR (anhydrodihydroartemisinin[Title/Abstract])) OR (artemisinin B[Title/Abstract])) OR (deoxoartemisinin[Title/Abstract])) OR (Artemisia annua[Title/Abstract])) OR (sulfadoxine-pyrimethamine-artesunate[Title/Abstract])) OR (artemisinic aldehyde delta11(13) reductase, Artemisia annua[Title/Abstract])) OR (ethyl 2-(4-(12-beta-artemisininoxy))phenoxylpropionate[Title/Abstract])) OR (SM905[Title/Abstract])) OR (amorpha-4,11-diene[Title/Abstract])) OR (Artemisone[Title/Abstract])) OR (99-357 compound[Title/Abstract])) OR (SM 486[Title/Abstract])) OR (Artemether[Title/Abstract])) OR (Artesunate[Title/Abstract])) OR (artesumycin[Title/Abstract])) |
| #5 | ((((((((((((((((((((((((((((((Artemisinins[Title/Abstract]) OR (artemisinin[Title/Abstract])) OR (arteannuin[Title/Abstract])) OR (qinghaosu[Title/Abstract])) OR (quing hau sau[Title/Abstract])) OR (quinghaosu[Title/Abstract])) OR (qing hao su[Title/Abstract])) OR (artemisininum[Title/Abstract])) OR (Artemisinine[Title/Abstract])) OR (artemisinin-naphthoquine combination[Title/Abstract])) OR (artemisinin G[Title/Abstract])) OR (artenimol[Title/Abstract])) OR (10-(2-(3-trifluoromethylphenylpiperazyl)ethyl)deoxoartemisinin[Title/Abstract])) OR (dihydroartemisinin beta-glucuronide[Title/Abstract])) OR (10-(4-trifluoromethylphenoxy)dihydroartemisinin[Title/Abstract])) OR (artemisinin trioxane alcohol[Title/Abstract])) OR (anhydrodihydroartemisinin[Title/Abstract])) OR (artemisinin B[Title/Abstract])) OR (deoxoartemisinin[Title/Abstract])) OR (Artemisia annua[Title/Abstract])) OR (sulfadoxine-pyrimethamine-artesunate[Title/Abstract])) OR (artemisinic aldehyde delta11(13) reductase, Artemisia annua[Title/Abstract])) OR (ethyl 2-(4-(12-beta-artemisininoxy))phenoxylpropionate[Title/Abstract])) OR (SM905[Title/Abstract])) OR (amorpha-4,11-diene[Title/Abstract])) OR (Artemisone[Title/Abstract])) OR (99-357 compound[Title/Abstract])) OR (SM 486[Title/Abstract])) OR (Artemether[Title/Abstract])) OR (Artesunate[Title/Abstract])) OR (artesumycin[Title/Abstract]) |
| #4 | (Artemisinins[MeSH Terms]) OR (artemisinin[MeSH Terms]) |
| #3 | (((((Depression[MeSH Terms]) OR (Depressive Disorder[MeSH Terms])) OR (Depression, Chemical[MeSH Terms])) OR (Depressive Disorder, Treatment-Resistant[MeSH Terms])) OR (Major Depressive Disorder[MeSH Terms])) OR ((((((((((((((((((((((((((((((((((((((((((((((((((((((((((((((((((((((((Depression[Title/Abstract]) OR (Depressive Disorder[Title/Abstract])) OR (Depression, Chemical[Title/Abstract])) OR (Depressive Disorder, Treatment-Resistant[Title/Abstract])) OR (Major Depressive Disorder[Title/Abstract])) OR (Depressive Symptoms[Title/Abstract])) OR (Depressive Symptom[Title/Abstract])) OR (Symptom, Depressive[Title/Abstract])) OR (Emotional Depression[Title/Abstract])) OR (Depression, Emotional[Title/Abstract])) OR (Depressive Disorders[Title/Abstract])) OR (Disorder, Depressive[Title/Abstract])) OR (Disorders, Depressive[Title/Abstract])) OR (Depression Disorder[Title/Abstract])) OR (Disorders, Depression[Title/Abstract])) OR (Neurosis, Depressive[Title/Abstract])) OR (Depressive Neuroses[Title/Abstract])) OR (Neuroses, Depressive[Title/Abstract])) OR (Depression, Endogenous[Title/Abstract])) OR (Depressions, Endogenous[Title/Abstract])) OR (Endogenous Depression[Title/Abstract])) OR (Melancholia[Title/Abstract])) OR (Unipolar Depression[Title/Abstract])) OR (Depression, Unipolar[Title/Abstract])) OR (Depressive Syndrome[Title/Abstract])) OR (Syndrome, Depressive[Title/Abstract])) OR (Syndromes, Depressive[Title/Abstract])) OR (Depression, Neurotic[Title/Abstract])) OR (Depressions, Neurotic[Title/Abstract])) OR (Neurotic Depression[Title/Abstract])) OR (Chemical Depression[Title/Abstract])) OR (Chemical Depressions[Title/Abstract])) OR (Depressions, Chemical[Title/Abstract])) OR (Depressive Disorders, Treatment-Resistant[Title/Abstract])) OR (Depressive Disorder, Treatment Resistant[Title/Abstract])) OR (Disorders, Treatment-Resistant Depressive[Title/Abstract])) OR (Disorder, Treatment-Resistant Depressive[Title/Abstract])) OR (Treatment-Resistant Depressive Disorder[Title/Abstract])) OR (Treatment-Resistant Depressive Disorders[Title/Abstract])) OR (Treatment Resistant Depression[Title/Abstract])) OR (Depressions, Treatment Resistant[Title/Abstract])) OR (Depression, Treatment Resistant[Title/Abstract])) OR (Resistant Depressions, Treatment[Title/Abstract])) OR (Resistant Depression, Treatment[Title/Abstract])) OR (Treatment Resistant Depressions[Title/Abstract])) OR (Refractory Depression[Title/Abstract])) OR (Depression, Refractory[Title/Abstract])) OR (Depressions, Refractory[Title/Abstract])) OR (Refractory Depressions[Title/Abstract])) OR (Therapy-Resistant Depression[Title/Abstract])) OR (Depressions, Therapy-Resistant[Title/Abstract])) OR (Depression, Therapy-Resistant[Title/Abstract])) OR (Therapy Resistant Depression[Title/Abstract])) OR (Therapy-Resistant Depressions[Title/Abstract])) OR (Depressive Disorders, Major[Title/Abstract])) OR (Disorder, Major Depressive[Title/Abstract])) OR (Disorders, Major Depressive[Title/Abstract])) OR (Major Depressive Disorders[Title/Abstract])) OR (Clinical Depression[Title/Abstract])) OR (Depressions, Clinical[Title/Abstract])) OR (Major Depression Disorder[Title/Abstract])) OR (Depression Disorders, Major[Title/Abstract])) OR (Disorders, Major Depression[Title/Abstract])) OR (Depressive Disorder, Major[Title/Abstract])) OR (Depression, Involutional[Title/Abstract])) OR (Depressions, Involutional[Title/Abstract])) OR (Melancholia, Involutional[Title/Abstract])) OR (Melancholias, Involutional[Title/Abstract])) OR (Psychosis, Involutional[Title/Abstract])) OR (Psychoses, Involutional[Title/Abstract])) OR (Paraphrenia, Involutional[Title/Abstract])) OR (Paraphrenias, Involutional[Title/Abstract])) |
| #2 | (((((((((((((((((((((((((((((((((((((((((((((((((((((((((((((((((((((((Depression[Title/Abstract]) OR (Depressive Disorder[Title/Abstract])) OR (Depression, Chemical[Title/Abstract])) OR (Depressive Disorder, Treatment-Resistant[Title/Abstract])) OR (Major Depressive Disorder[Title/Abstract])) OR (Depressive Symptoms[Title/Abstract])) OR (Depressive Symptom[Title/Abstract])) OR (Symptom, Depressive[Title/Abstract])) OR (Emotional Depression[Title/Abstract])) OR (Depression, Emotional[Title/Abstract])) OR (Depressive Disorders[Title/Abstract])) OR (Disorder, Depressive[Title/Abstract])) OR (Disorders, Depressive[Title/Abstract])) OR (Depression Disorder[Title/Abstract])) OR (Disorders, Depression[Title/Abstract])) OR (Neurosis, Depressive[Title/Abstract])) OR (Depressive Neuroses[Title/Abstract])) OR (Neuroses, Depressive[Title/Abstract])) OR (Depression, Endogenous[Title/Abstract])) OR (Depressions, Endogenous[Title/Abstract])) OR (Endogenous Depression[Title/Abstract])) OR (Melancholia[Title/Abstract])) OR (Unipolar Depression[Title/Abstract])) OR (Depression, Unipolar[Title/Abstract])) OR (Depressive Syndrome[Title/Abstract])) OR (Syndrome, Depressive[Title/Abstract])) OR (Syndromes, Depressive[Title/Abstract])) OR (Depression, Neurotic[Title/Abstract])) OR (Depressions, Neurotic[Title/Abstract])) OR (Neurotic Depression[Title/Abstract])) OR (Chemical Depression[Title/Abstract])) OR (Chemical Depressions[Title/Abstract])) OR (Depressions, Chemical[Title/Abstract])) OR (Depressive Disorders, Treatment-Resistant[Title/Abstract])) OR (Depressive Disorder, Treatment Resistant[Title/Abstract])) OR (Disorders, Treatment-Resistant Depressive[Title/Abstract])) OR (Disorder, Treatment-Resistant Depressive[Title/Abstract])) OR (Treatment-Resistant Depressive Disorder[Title/Abstract])) OR (Treatment-Resistant Depressive Disorders[Title/Abstract])) OR (Treatment Resistant Depression[Title/Abstract])) OR (Depressions, Treatment Resistant[Title/Abstract])) OR (Depression, Treatment Resistant[Title/Abstract])) OR (Resistant Depressions, Treatment[Title/Abstract])) OR (Resistant Depression, Treatment[Title/Abstract])) OR (Treatment Resistant Depressions[Title/Abstract])) OR (Refractory Depression[Title/Abstract])) OR (Depression, Refractory[Title/Abstract])) OR (Depressions, Refractory[Title/Abstract])) OR (Refractory Depressions[Title/Abstract])) OR (Therapy-Resistant Depression[Title/Abstract])) OR (Depressions, Therapy-Resistant[Title/Abstract])) OR (Depression, Therapy-Resistant[Title/Abstract])) OR (Therapy Resistant Depression[Title/Abstract])) OR (Therapy-Resistant Depressions[Title/Abstract])) OR (Depressive Disorders, Major[Title/Abstract])) OR (Disorder, Major Depressive[Title/Abstract])) OR (Disorders, Major Depressive[Title/Abstract])) OR (Major Depressive Disorders[Title/Abstract])) OR (Clinical Depression[Title/Abstract])) OR (Depressions, Clinical[Title/Abstract])) OR (Major Depression Disorder[Title/Abstract])) OR (Depression Disorders, Major[Title/Abstract])) OR (Disorders, Major Depression[Title/Abstract])) OR (Depressive Disorder, Major[Title/Abstract])) OR (Depression, Involutional[Title/Abstract])) OR (Depressions, Involutional[Title/Abstract])) OR (Melancholia, Involutional[Title/Abstract])) OR (Melancholias, Involutional[Title/Abstract])) OR (Psychosis, Involutional[Title/Abstract])) OR (Psychoses, Involutional[Title/Abstract])) OR (Paraphrenia, Involutional[Title/Abstract])) OR (Paraphrenias, Involutional[Title/Abstract]) |
| #1 | ((((Depression[MeSH Terms]) OR (Depressive Disorder[MeSH Terms])) OR (Depression, Chemical[MeSH Terms])) OR (Depressive Disorder, Treatment-Resistant[MeSH Terms])) OR (Major Depressive Disorder[MeSH Terms]) |
| **Embase** |  |
| #3 | #1 AND #2 |
| #2 | artemisinins'/exp OR artemisinins OR 'artemisinin'/exp OR artemisinin OR 'arteannuin'/exp OR arteannuin OR 'qinghaosu'/exp OR qinghaosu OR 'quing hau sau' OR (quing AND hau AND sau) OR 'quinghaosu'/exp OR quinghaosu OR huanghuahaosu OR 'qing hao su' OR (qing AND hao AND su) OR artemisininum OR 'artemisinine'/exp OR artemisinine OR 'artemisinin-naphthoquine combination' OR ('artemisinin naphthoquine' AND combination) OR 'artemisinin g' OR (('artemisinin'/exp OR artemisinin) AND g) OR '3 hydroxydeoxyartemisinin' OR 'artenimol'/exp OR artenimol OR 'dihydroartemisinin beta-glucuronide' OR (('dihydroartemisinin'/exp OR dihydroartemisinin) AND ('beta glucuronide'/exp OR 'beta glucuronide')) OR '10 phenoxydihydroartemisinin' OR carboxypropyldeoxoartemisinine OR 'artemisinin trioxane alcohol' OR (('artemisinin'/exp OR artemisinin) AND ('trioxane'/exp OR trioxane) AND ('alcohol'/exp OR alcohol)) OR 'anhydrodihydroartemisinin'/exp OR anhydrodihydroartemisinin OR 'artemisinin b'/exp OR 'artemisinin b' OR (('artemisinin'/exp OR artemisinin) AND b) OR 'deoxoartemisinin'/exp OR deoxoartemisinin OR 'artemisia annua'/exp OR 'artemisia annua' OR (('artemisia'/exp OR artemisia) AND annua) OR 'sulfadoxine pyrimethamine artesunate'/exp OR 'sulfadoxine pyrimethamine artesunate' OR sm905 OR '7 hydroxyartemisinin' OR '5 hydroxyartemisinin' OR 'amorpha 4,11 diene' OR 'artemisone'/exp OR artemisone OR '99-357 compound' OR ('99 357' AND ('compound'/exp OR compound)) OR 'sm 486' OR (sm AND 486) OR 'artemether'/exp OR artemether OR 'artesunate'/exp OR artesunate OR artesumycin |
| #1 | depression'/exp OR depression OR 'depressive disorder'/exp OR 'depressive disorder' OR (depressive AND ('disorder'/exp OR disorder)) OR 'depression, chemical'/exp OR 'depression, chemical' OR (('depression,'/exp OR depression,) AND chemical) OR 'depressive disorder, treatment-resistant'/exp OR 'depressive disorder, treatment-resistant' OR (depressive AND ('disorder,'/exp OR disorder,) AND 'treatment resistant') OR 'major depressive disorder'/exp OR 'major depressive disorder' OR (major AND depressive AND ('disorder'/exp OR disorder)) OR 'depressive symptoms'/exp OR 'depressive symptoms' OR (depressive AND ('symptoms'/exp OR symptoms)) OR 'depressive symptom'/exp OR 'depressive symptom' OR (depressive AND ('symptom'/exp OR symptom)) OR 'symptom, depressive' OR (('symptom,'/exp OR symptom,) AND depressive) OR 'emotional depression' OR (emotional AND ('depression'/exp OR depression)) OR 'depression, emotional' OR (('depression,'/exp OR depression,) AND emotional) OR 'depressive disorders' OR (depressive AND ('disorders'/exp OR disorders)) OR 'disorder, depressive' OR (('disorder,'/exp OR disorder,) AND depressive) OR 'disorders, depressive' OR (('disorders,'/exp OR disorders,) AND depressive) OR 'depression disorder' OR (('depression'/exp OR depression) AND ('disorder'/exp OR disorder)) OR 'disorders, depression' OR (('disorders,'/exp OR disorders,) AND ('depression'/exp OR depression)) OR 'neurosis, depressive' OR (('neurosis,'/exp OR neurosis,) AND depressive) OR 'depressive neuroses' OR (depressive AND ('neuroses'/exp OR neuroses)) OR 'neuroses, depressive' OR (neuroses, AND depressive) OR 'depression, endogenous'/exp OR 'depression, endogenous' OR (('depression,'/exp OR depression,) AND endogenous) OR 'depressions, endogenous' OR (depressions, AND endogenous) OR 'endogenous depression'/exp OR 'endogenous depression' OR (endogenous AND ('depression'/exp OR depression)) OR 'melancholia'/exp OR melancholia OR 'unipolar depression'/exp OR 'unipolar depression' OR (unipolar AND ('depression'/exp OR depression)) OR 'depression, unipolar'/exp OR 'depression, unipolar' OR (('depression,'/exp OR depression,) AND unipolar) OR 'depressive syndrome'/exp OR 'depressive syndrome' OR (depressive AND ('syndrome'/exp OR syndrome)) OR 'syndrome, depressive' OR (('syndrome,'/exp OR syndrome,) AND depressive) OR 'syndromes, depressive' OR (syndromes, AND depressive) OR 'depression, neurotic'/exp OR 'depression, neurotic' OR (('depression,'/exp OR depression,) AND neurotic) OR 'depressions, neurotic' OR (depressions, AND neurotic) OR 'neurotic depression'/exp OR 'neurotic depression' OR (neurotic AND ('depression'/exp OR depression)) OR 'chemical depression' OR (chemical AND ('depression'/exp OR depression)) OR 'chemical depressions' OR (chemical AND depressions) OR 'depressions, chemical' OR (depressions, AND chemical) OR 'depressive disorders, treatment-resistant' OR (depressive AND ('disorders,'/exp OR disorders,) AND 'treatment resistant') OR 'depressive disorder, treatment resistant'/exp OR 'depressive disorder, treatment resistant' OR (depressive AND ('disorder,'/exp OR disorder,) AND ('treatment'/exp OR treatment) AND resistant) OR 'disorders, treatment-resistant depressive' OR (('disorders,'/exp OR disorders,) AND 'treatment resistant' AND depressive) OR 'disorder, treatment-resistant depressive' OR (('disorder,'/exp OR disorder,) AND 'treatment resistant' AND depressive) OR 'treatment-resistant depressive disorder'/exp OR 'treatment-resistant depressive disorder' OR ('treatment resistant' AND depressive AND ('disorder'/exp OR disorder)) OR 'treatment-resistant depressive disorders' OR ('treatment resistant' AND depressive AND ('disorders'/exp OR disorders)) OR 'treatment resistant depression'/exp OR 'treatment resistant depression' OR (('treatment'/exp OR treatment) AND resistant AND ('depression'/exp OR depression)) OR 'depressions, treatment resistant' OR (depressions, AND ('treatment'/exp OR treatment) AND resistant) OR 'depression, treatment resistant' OR (('depression,'/exp OR depression,) AND ('treatment'/exp OR treatment) AND resistant) OR 'resistant depressions, treatment' OR (resistant AND depressions, AND ('treatment'/exp OR treatment)) OR 'resistant depression, treatment' OR (resistant AND ('depression,'/exp OR depression,) AND ('treatment'/exp OR treatment)) OR 'treatment resistant depressions' OR (('treatment'/exp OR treatment) AND resistant AND depressions) OR 'refractory depression'/exp OR 'refractory depression' OR (refractory AND ('depression'/exp OR depression)) OR 'depression, refractory' OR (('depression,'/exp OR depression,) AND refractory) OR 'depressions, refractory' OR (depressions, AND refractory) OR 'refractory depressions' OR (refractory AND depressions) OR 'therapy-resistant depression'/exp OR 'therapy-resistant depression' OR ('therapy resistant' AND ('depression'/exp OR depression)) OR 'depressions, therapy-resistant' OR (depressions, AND 'therapy resistant') OR 'depression, therapy-resistant' OR (('depression,'/exp OR depression,) AND 'therapy resistant') OR 'therapy resistant depression'/exp OR 'therapy resistant depression' OR (('therapy'/exp OR therapy) AND resistant AND ('depression'/exp OR depression)) OR 'therapy-resistant depressions' OR ('therapy resistant' AND depressions) OR 'depressive disorders, major' OR (depressive AND ('disorders,'/exp OR disorders,) AND major) OR 'disorder, major depressive' OR (('disorder,'/exp OR disorder,) AND major AND depressive) OR 'disorders, major depressive' OR (('disorders,'/exp OR disorders,) AND major AND depressive) OR 'major depressive disorders' OR (major AND depressive AND ('disorders'/exp OR disorders)) OR 'clinical depression'/exp OR 'clinical depression' OR (('clinical'/exp OR clinical) AND ('depression'/exp OR depression)) OR 'depressions, clinical' OR (depressions, AND ('clinical'/exp OR clinical)) OR 'major depression disorder' OR (major AND ('depression'/exp OR depression) AND ('disorder'/exp OR disorder)) OR 'depression disorders, major' OR (('depression'/exp OR depression) AND ('disorders,'/exp OR disorders,) AND major) OR 'disorders, major depression' OR (('disorders,'/exp OR disorders,) AND major AND ('depression'/exp OR depression)) OR 'depressive disorder, major'/exp OR 'depressive disorder, major' OR (depressive AND ('disorder,'/exp OR disorder,) AND major) OR 'depression, involutional'/exp OR 'depression, involutional' OR (('depression,'/exp OR depression,) AND involutional) OR 'depressions, involutional' OR (depressions, AND involutional) OR 'melancholia, involutional' OR (('melancholia,'/exp OR melancholia,) AND involutional) OR 'melancholias, involutional' OR (melancholias, AND involutional) OR 'psychosis, involutional' OR (('psychosis,'/exp OR psychosis,) AND involutional) OR 'psychoses, involutional' OR (psychoses, AND involutional) OR 'paraphrenia, involutional' OR (('paraphrenia,'/exp OR paraphrenia,) AND involutional) OR 'paraphrenias, involutional' OR (paraphrenias, AND involutional) |
| **Web of science** |  |
| #1 | Depression (Topic) or Depressive Disorder (Topic) or Depression, Chemical (Topic) or Depressive Disorder, Treatment-Resistant (Topic) or Major Depressive Disorder (Topic) or Depressive Symptoms (Topic) or Depressive Symptom (Topic) or Symptom, Depressive (Topic) or Emotional Depression (Topic) or Depression, Emotional (Topic) or Depressive Disorders (Topic) or Disorder, Depressive (Topic) or Disorders, Depressive (Topic) or Depression Disorder (Topic) or Disorders, Depression (Topic) or Neurosis, Depressive (Topic) or Depressive Neuroses (Topic) or Neuroses, Depressive (Topic) or Depression, Endogenous (Topic) or Depressions, Endogenous (Topic) or Endogenous Depression (Topic) or Melancholia (Topic) or Unipolar Depression (Topic) or Depression, Unipolar (Topic) or Depressive Syndrome (Topic) or Syndrome, Depressive (Topic) or Syndromes, Depressive (Topic) or Depression, Neurotic (Topic) or Depressions, Neurotic (Topic) or Neurotic Depression (Topic) or Chemical Depression (Topic) or Chemical Depressions (Topic) or Depressions, Chemical (Topic) or Depressive Disorders, Treatment-Resistant (Topic) or Depressive Disorder, Treatment Resistant (Topic) or Disorders, Treatment-Resistant Depressive (Topic) or Disorder, Treatment-Resistant Depressive (Topic) or Treatment-Resistant Depressive Disorder (Topic) or Treatment-Resistant Depressive Disorders (Topic) or Treatment Resistant Depression (Topic) or Depressions, Treatment Resistant (Topic) or Depression, Treatment Resistant (Topic) or Resistant Depressions, Treatment (Topic) or Resistant Depression, Treatment (Topic) or Treatment Resistant Depressions (Topic) or Refractory Depression (Topic) or Depression, Refractory (Topic) or Depressions, Refractory (Topic) or Refractory Depressions (Topic) or Therapy-Resistant Depression (Topic) or Depressions, Therapy-Resistant (Topic) or Depression, Therapy-Resistant (Topic) or Therapy Resistant Depression (Topic) or Therapy-Resistant Depressions (Topic) or Depressive Disorders, Major (Topic) or Disorder, Major Depressive (Topic) or Disorders, Major Depressive (Topic) or Major Depressive Disorders (Topic) or Clinical Depression (Topic) or Depressions, Clinical (Topic) or Major Depression Disorder (Topic) or Depression Disorders, Major (Topic) or Disorders, Major Depression (Topic) or Depressive Disorder, Major (Topic) or Depression, Involutional (Topic) or Depressions, Involutional (Topic) or Melancholia, Involutional (Topic) or Melancholias, Involutional (Topic) or Psychosis, Involutional (Topic) or Psychoses, Involutional (Topic) or Paraphrenia, Involutional (Topic) or Paraphrenias, Involutional (Topic) |
| #2 | Artemisinins (Topic) or artemisinin (Topic) or arteannuin (Topic) or qinghaosu (Topic) or quing hau sau (Topic) or quinghaosu (Topic) or huanghuahaosu (Topic) or qing hao su (Topic) or artemisininum (Topic) or Artemisinine (Topic) or artemisinin-naphthoquine combination (Topic) or artemisinin G (Topic) or 3-hydroxydeoxyartemisinin (Topic) or artenimol (Topic) or 10-(2-(3-trifluoromethylphenylpiperazyl)ethyl)deoxoartemisinin (Topic) or dihydroartemisinin beta-glucuronide (Topic) or 10-(4-trifluoromethylphenoxy)dihydroartemisinin (Topic) or 10-phenoxydihydroartemisinin (Topic) or carboxypropyldeoxoartemisinine (Topic) or artemisinin trioxane alcohol (Topic) or anhydrodihydroartemisinin (Topic) or artemisinin B (Topic) or deoxoartemisinin (Topic) or Artemisia annua (Topic) or sulfadoxine-pyrimethamine-artesunate (Topic) or artemisinic aldehyde delta11(13) reductase, Artemisia annua (Topic) or ethyl 2-(4-(12-beta-artemisininoxy))phenoxylpropionate (Topic) or SM905 (Topic) or 7-hydroxyartemisinin (Topic) or 5-Hydroxyartemisinin (Topic) or amorpha-4,11-diene (Topic) or Artemisone (Topic) or 99-357 compound (Topic) or octahydro-3,6-dimethyl-3,12-epoxy-9-(3'-(3-trifluoromethylphenyl)propyl)-12H-pyrano(4,3-j)-1,2-benzodioxepin (Topic) or octahydro-3,6-dimethyl-3,12-epoxy-9-(3'-(3,4-dichlorphenyl)propyl)-12H-pyrano(4,3-j)-1,2-benzodioxepin (Topic) or SM 486 (Topic) or Artemether (Topic) or Artesunate (Topic) or artesumycin (Topic) |
| #3 | #1 AND #2 |
| **CNKI** |  |
|  | (SU %= '青蒿' OR SU %= '青蒿素') AND (SU %= '=抑郁' OR SU %= '抑郁情绪' OR SU %= '抑郁症' ) |
